# Far-red light in early growth stages boosts lettuce biomass and preserves anthocyanins

**DOI:** 10.1093/aob/mcag031

**Published:** 2026-03-09

**Authors:** Christopher P Levine, Keiichiro Tanigawa, Yu Wakabayashi, Wei Guo, Yuchen Qu, Ichiro Terashima, Wataru Yamori

**Affiliations:** Graduate School of Agricultural and Life Sciences, The University of Tokyo, Nishitokyo, Tokyo 188-0002, Japan; Graduate School of Agricultural and Life Sciences, The University of Tokyo, Nishitokyo, Tokyo 188-0002, Japan; Graduate School of Agricultural and Life Sciences, The University of Tokyo, Nishitokyo, Tokyo 188-0002, Japan; Graduate School of Agricultural and Life Sciences, The University of Tokyo, Nishitokyo, Tokyo 188-0002, Japan; Graduate School of Agricultural and Life Sciences, The University of Tokyo, Nishitokyo, Tokyo 188-0002, Japan; Graduate School of Agricultural and Life Sciences, The University of Tokyo, Nishitokyo, Tokyo 188-0002, Japan; Institute of Molecular Biology, National Chung Hsing University, South District, Taichung 40227, Taiwan; Graduate School of Agricultural and Life Sciences, The University of Tokyo, Nishitokyo, Tokyo 188-0002, Japan

**Keywords:** Far-red, far-red biology, lettuce (*Lactuca sativa*), plant factory, LED, anthocyanin, photosynthesis, plant shape

## Abstract

**Background and Aims:**

Light plays a dual role in plants, serving as both an energy source and a regulator of development from seedling to senescence. Recently, far-red (FR) radiation has gained attention in the controlled environment agriculture (CEA) science and grower community for its potential to enhance yield through canopy expansion and improved light capture, contributing positively to photosynthesis. This study explores how supplementary FR light promotes lettuce growth and morphology across weekly intervals as well as analysing photosynthetic parameters, pigment accumulation and anthocyanin gene expression.

**Methods:**

Red leaf lettuce (*Lactuca sativa* ‘Red Fire’) was grown in a commercial plant factory with artificial light for 6 weeks. White (W) light, 5000 K, was maintained at 300 μmol m^−2^ s^−1^, and FR, when supplemented, was added at 100 μmol m^−2^ s^−1^ in addition to the 300 μmol m^−2^ s^−1^ of W light. Four lighting treatments were tested under a 16-h photoperiod: (1) W for all 6 weeks (treatment W), (2) 4 weeks of W followed by 2 weeks of supplementary FR (W to W + FR), (3) 4 weeks of FR supplementation followed by 2 weeks of only W (W + FR to W), and (4) W + FR for all 6 weeks (W + FR).

**Key Results:**

The shoot dry weight after 6 weeks in W + FR, W + FR to W and W to W + FR was greater than that in W. Both W + FR and W + FR to W showed a tendency for greater canopy expansion compared with W as well as W to W + FR. There were no significant differences in stomatal conductance among the treatments. On the other hand, in both W and W + FR to W plants the CO_2_ assimilation rates were enhanced when FR light was supplemented during measurement, compared with when FR was not provided. Anthocyanin accumulation was greater in both W and W + FR to W, consistent with the expression of key genes involved in the anthocyanin biosynthesis pathway, including anthocyanin synthase (*ANS*), flavanone 3-hydroxylase (*F3H*) and dihydroflavonol 4-reductase (*DFR*).

**Conclusions:**

This study demonstrates that FR supplementation during the early growth stages of lettuce promotes biomass accumulation by enhancing both canopy expansion and photosynthetic activity, while maintaining high levels of functional compounds such as anthocyanins.

## INTRODUCTION

The global food system accounts for 30 % of annual greenhouse gas emissions, with 19 % attributed to food transport, particularly for refrigerated fruits and vegetables (Li *et al*., 2022). Local crop production currently fulfils less than one-third of global food demand (Kinnunen *et al*., 2020; Li *et al*., 2022). Mitigating environmental impact requires prioritizing local food production, particularly in affluent nations, where food-mile emissions are a significant contributor. Controlled environment agriculture (CEA) presents a promising approach for enhancing local food self-sufficiency, especially in light of supply chain disruptions caused by geopolitical instability and global crises such as pandemics (Furuta *et al*., 2025; Qiu *et al*., 2025; Takano *et al*., 2025). However, CEA faces several obstacles, including challenges related to profitability, energy efficiency, supportive public policy and consumer acceptance (van Delden *et al*., 2021).

Light is one of the most important environmental factors regulating plant growth and phytochemical accumulation in artificial light plant factories (Kopsell and Kopsell, 2008; Pérez-Balibrea *et al*., 2008; Matsuura *et al*., 2013). Because chlorophyll absorbs red and blue wavelengths most efficiently, the effects of red and blue light on lettuce growth, photosynthesis and quality traits have been extensively characterized under controlled environments (Ohtake *et al*., 2018, 2021; Kaiser *et al*., 2019; Li *et al*., 2025). Blue light applied late in production increases anthocyanins, enhancing colour and quality in red leaf lettuce (Owen *et al*., 2015; Kelly *et al*, 2023; Zhu *et al*., 2024*a*). It has also been shown that supplementary FR light can decrease anthocyanin concentration but promote leaf extension and plant growth in lettuce (Gommers *et al*., 2013; He *et al*., 2021). Ultraviolet (UV) light also has been shown to stimulate the accumulation of secondary metabolites, including anthocyanins, carotenoids and chlorophylls, primarily through stress and signalling-mediated responses rather than direct contributions to photosynthesis (Vergeer *et al*., 1995; Keller *et al*., 1998; Tsormpatsidis *et al*., 2008; Chen *et al*., 2019; He *et al*., 2021).

Anthocyanin accumulation in red leaf lettuce is also regulated by the coordinated expression of genes in the flavonoid biosynthetic pathway, many of which are highly responsive to light quality. Goto *et al*. (2016) demonstrated that short-term UV-B exposure significantly increased the expression of key anthocyanin biosynthetic genes, including *CHS* (chalcone synthase), *F3H* (flavanone 3-hydroxylase), *DFR* (dihydroflavonol 4-reductase), *ANS* (anthocyanidin synthase) and *UFGT* (UDP-glucose:flavonoid 3-*O*-glucosyltransferase), which collectively govern the synthesis and stabilization of anthocyanins in lettuce leaves. Light manipulation is a well-established environmental strategy for inducing these genes and enhancing antioxidant capacity in leafy vegetables. Together, these findings indicate that spectral cues regulate anthocyanin accumulation at the transcriptional level and provide a mechanistic framework for evaluating how alternative light treatments influence anthocyanin biosynthesis in controlled environment systems. However, despite this extensive knowledge of red, blue and UV radiation, the role of FR light in balancing biomass production and phytochemical accumulation under commercially relevant plant factory conditions remains less clearly defined.

There is also no universally optimal FR or broader spectral lighting recipe (e.g. 300–800 nm) for plant cultivation in plant factories, as light requirements vary depending on many factors (Piovene *et al*., 2015; Thilini Deepashika Perera *et al*., 2022; Barbieri *et al*., 2023). Furthermore, it has been stated that every vertical farming system is different (Carpineti *et al*., 2024), and variability exists between research and production modules, thus making consolidated findings from different studies not straightforward. This further supports the need to conduct FR lettuce studies under commercially realistic conditions in order to clarify whether the treatments result in changes rather than in experimental conditions that are unrealistic in commercial settings.

Supplementing plant lighting with FR radiation has gained considerable attention in recent years. It is well known that FR induces shade avoidance responses, leading to stem elongation, while shade tolerance mechanisms promote leaf expansion in various but not all plants (Owen *et al*., 2015; Zhen *et al*., 2020; Kusuma *et al*., 2023; Van de Velde *et al*., 2023; Skabelund *et al*., 2025). Studies indicate that FR photon supplementation enhances plant biomass accumulation by promoting leaf expansion, leading to increased light capture (Li and Kubota, 2009; Park and Runkle, 2017; Meng and Runkle, 2019; Zhen *et al*., 2020). Exposure to FR light triggers the conversion of phytochromes from their active (Pfr) to inactive (Pr) forms, with peak absorption occurring at 730 nm. In contrast, red light (670 nm) reverts phytochromes to their active state (Sharrock, 2008; Kaiser *et al*., 2024). Notably, the photon flux density (PFD) of FR photons (∼700–750 nm) is 18–19 % of the photosynthetic PFD (PPFD; 400–700 nm) under direct unfiltered sunlight (ASTM standard, 2020). However, most horticultural LED fixtures for CEA emit less than 2 % as FR except for some lights designed specifically to regulate flowering (Runkle, 2022).

Far-red LEDs also often exhibit a broad emission spectrum, with small amounts of red light present near the 700-nm range due to their natural bandwidth. Achieving a sharp spectral cut-off between red and FR regions is challenging without the use of laser diodes or optical filters. Therefore, future developments in FR LEDs with narrower bandwidths or peaks beyond ∼730 nm may help eliminate red light contamination. Recognizing these spectral overlaps is important when interpreting plant responses and designing lighting systems for CEA research. Furthermore, productivity can be enhanced by controlling the ratio of red to blue light (Lim *et al*., 2023).

Far-red supplementation has also been found to reduce anthocyanin concentration in lettuce cultivars, which may affect consumer purchasing preferences (Van Brenk *et al*., 2024). Additionally, higher FR intensities in choy sum (*Brassica rapa* subsp*. chinensis* var*. parachinensis*) have been linked to a decrease in total carotenoid content (Zou *et al*., 2024), while in red butterhead lettuce FR supplementation significantly reduced concentrations of calcium, magnesium, manganese and phosphorus (Van Brenk *et al*., 2024). While FR radiation can enhance biomass accumulation, it may cause dilution effects, reducing concentrations of certain compounds per unit leaf area or mass without lowering total contents per plant. For instance, Kelly and Runkle (2024) found that partial replacement of red with FR light increased leaf area, diluting anthocyanin concentrations and pigmentation. This highlights the need to consider measurement units and balance trade-offs when applying FR effectively.

Beyond mineral and phytochemical accumulation, another critical aspect influencing the effects of FR on plant growth is the practical context of dense growing environments, where overlapping leaves play a major role. Zhen and Bugbee (2020) reported equivalent canopy photosynthesis with partial FR substitution across 14 species. However, their plants were grown and acclimated in a glass-covered greenhouse under unspecified high or low light treatments, from which subsequent short-term photosynthetic parameters were collected. Since plant photosynthetic responses can vary with light acclimation, particularly under prolonged exposure, the generalization of these results to plant factories with 100 % artificial light is uncertain. Notably, Jeong *et al*. (2024) found that 20 % FR substitution significantly reduced net photosynthesis in lettuce leaves acclimated to FR, and Zou *et al*. (2019) observed significant declines in photosynthetic rates after 14–17 d of FR acclimation.

Overall, the objective of this research is to evaluate FR radiation supplementation under practical commercial plant factory with artificial light (PFAL) conditions. Specifically, we assess the impact of FR supplementation on a red leaf lettuce cultivar (*Lactuca sativa* ‘Red Fire’) grown for 6 weeks under a background of 300 µmol m^−2^ s^−1^ 5000-K white light in a commercial plant factory with artificial light (PFAL). The 5000-K white LED fixtures were pre-installed as the standard operational lighting system of the commercial facility and did not allow independent spectral tuning. Accordingly, this study evaluates FR supplementation within the constraints of an existing commercial PFAL system, rather than aiming to define an optimal spectral recipe under fully customizable research conditions. Parameters of interest focused on yield, morphology, plant architecture, anthocyanin gene expression, stomatal conductance, photosynthesis and metabolite profiles across two defined treatment phases, with weekly measurements capturing morphological changes over time. We hypothesize that supplementary FR radiation during the early growth stages (from the beginning of week 1 to the end of week 4) increases lettuce biomass via canopy expansion and photosynthetic performance, without reducing anthocyanin accumulation.

## MATERIALS AND METHODS

### Light setup

**Fig. 1.**
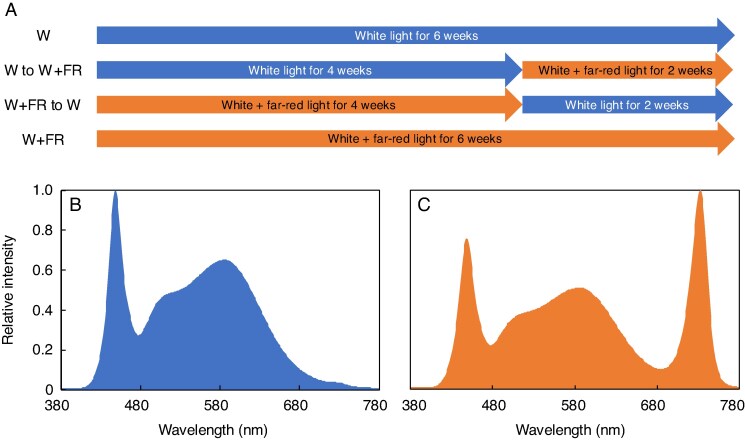
(A) The four experimental light treatments, each consisting of a 16-h photoperiod followed by an 8-h dark period: (1) white (W) light only for 6 weeks as the control, (2) 4 weeks of W light followed by 2 weeks of supplementary FR (W to W + FR), (3) 4 weeks of FR supplementation followed by 2 weeks of W light (W + FR to W), and (4) FR supplementation for 6 weeks (W + FR). (B, C) Representative spectral photon distributions of W and W+FR lighting treatments. (B) 5000 K W LED containing 300 µmol m^−2^ s^−1^ of 400–700 nm photosynthetically active radiation (PAR). (C) W + FR treatment containing 300 µmol m^−2^ s^−1^ PAR and 100 µmol m^−2^ s^−1^ of FR photons.

The experiment was conducted in a commercial PFAL in Tanashi, Nishitokyo, Japan (Plants Laboratory Inc., Tokyo, Japan). White LED lights (5000 K; TecoG II-40N2-5-23, Toshin Electric Co., Ltd., Osaka, Japan) were used along with FR LED lights (NK system 11A-Z20E4073, Nippon Medical and Chemical Instruments Co., Ltd., Osaka, Japan; see the wavelength in Fig. 1B, C). The white and FR LED lights were installed in a manner that kept light distribution as homogenous as possible (Supplementary Data Fig. S1). The LED lights were not dimmable. Spectral characteristics were measured at a fixed height in 36 spots placed under the growing canopy using an LA-105 Light Analyzer (NK system LA-105, Nippon Medical and Chemical Instruments Co., Ltd., Osaka, Japan) to map out light distribution across the whole cultivation zone (300 mm × 1200 mm). The four lighting treatments include a 16-h photoperiod of (1) 6 weeks of white light (control) (W), (2) 4 weeks of white light followed by 2 weeks of FR light (W to W + FR), (3) FR light for 4 weeks followed by 2 weeks of white light (W + FR to W), and (4) continuous FR light for the entire 6 weeks (W + FR) (Fig. 1A). The first 4 weeks only had two treatments, which consisted of only (W) and (W + FR). Starting from week 5, we had W, W + FR, W to W + FR and W + FR to W treatments which is why we only have treatments 1 (W) and 4 (W + FR) for the first 4 weeks in Fig. 3. The 4-week transplant point reflects standard practice used in the operational system of an established commercial head lettuce PFAL in Japan, where transplanting from the second-highest density is required due to plant growth canopy expansion. While the 4-week transplant point aligned well with our system’s specific combination of cultivar, lighting treatments, temperature set points and tray densities, we acknowledge that different cultivars or system configurations may warrant alternative transplant timings. White light (5000 K) was provided at 300 μmol m^−2^ s^−1^, and when additional FR was supplemented it was applied at 100 μmol m^−2^ s^−1^ (Fig. 1C, D). Table 1 provides a detailed description of the light intensity and spectrum measured ∼40 cm from the light. The light calculation of emitted light is not the same as light delivered to the plant canopy. Over the course of the experiment, light intensity applied to the plant was maintained constant regardless of the growth stage of the plants. Therefore, as the plants grew towards the light bars, they received higher light intensity. We also measured PPFD at 14 locations under the lighting canopy using a quantum sensor (Model MQ-200, Apogee Instruments, Inc., Logan, UT, USA) positioned ∼20 cm above the plant canopy, corresponding to the expected height of a mature canopy. Before harvest, the maximum light intensity at the top of the canopy was ∼415 ± 17 µmol m^−2^ s^−1^ (s.e.). The spectrum homogeneity was not affected by the change in light intensity, and the light was thoroughly mapped to ensure consistency between treatments.

**Table 1.** Spectral characteristics ± standard error of W and W + FR lighting treatments emitted by 5000 K white and FR LEDs.

Characteristic		W	W + FR
Integrated PFD (μmol m^−2^ d^−1^)			
PPFD	400–700 nm	299.3 ± 3.4	299.3 ± 2.5
FR-PFD	700–780 nm	5.5 ± 0.1	90.3 ± 1.5
Total PFD (TPFD)	400–780 nm	305.1 ± 3.4	389.8 ± 1.9
Yield PFD (YPFD)^1^	380–780 nm	255.9 ± 2.9	281.9 ± 3.4
Daily light integral (DLI) (mol m^−2^ d^−1^)			
PAR DLI^2^	400–700 nm	18.3 ± 0.5	18.4 ± 0.6
FR DLI^3^	700–780 nm	0.4 ± 0.0	5.0 ± 2.3
TPFD DLI^4^	400–780 nm	18.8 ± 0.5	24.6 ± 2.6
YPFD DLI^5^	380–780 nm	15.7 ± 0.4	17.4 ± 0.6
Waveband percentage of TPFD			
Blue	400–500 nm	24.6 ± 0.1 %	20.5 ± 2.0 %
Green	500–600 nm	45.9 ± 0.0 %	37.9 ± 4.0 %
Red	600–700 nm	26.9 ± 0.1 %	22.9 ± 2.1 %
Far-red	700–780 nm	2.4 ± 0.0 %	18.6 ± 8.1 %
Waveband percentage of PPFD			
Blue	400–500 nm	25.3 ± 0.1 %	25.2 ± 0.0 %
Green	500–600 nm	47.1 ± 0.0 %	46.6 ± 0.3 %
Red	600–700 nm	27.6 ± 0.1 %	28.2 % ± 0.3 %
Far-red	700–780 nm	2.4 % ± 0.0 %	25.1 % ± 11.4 %

^1^YPFD (μmol m^−2^ s^−1^, 380–780 nm): biologically weighted photon flux calculated as YPFD = *Σ*[*λ*=380 to 780] SPD(*λ*) × RQE(*λ*) where SPD is spectral photon flux density (μmol m^−2^ s^−1^ nm^−1^) and RQE is relative quantum efficiency. The RQE spectrum is from Sager *et al*. (1988), originally tabulated at 2-nm intervals and linearly interpolated to 1-nm resolution to match the measurement data. This metric reflects photosynthetically weighted photon flux from 380 to 780 nm.

^2^PPFD × 16-h photoperiod.

^3^FR PFD × 16-h photoperiod.

^4^TPFD × 16-h photoperiod.

^5^YPFD × 16-h photoperiod.

### Plant materials and growth conditions

Pelleted seeds of ‘Red Fire’ red leaf lettuce (*Lactuca sativa*, Takii Seed Co., Kyoto, Japan) were used. Urethane cubes were hydrated with reverse osmosis water and hydroponic fertilizer to an electrical conductivity of 1.50 ± 0.05 dS m^−1^ (GG liquid A and B stock solutions, Green Green Co., Ltd, Fukuoka, Japan). The environmental conditions in the PFAL were controlled and logged in real time, maintaining an air temperature of 22 ± 2 °C (day/night) with a relative humidity of 63 ± 6 %. These densities were selected primarily to emulate production practices of a commercial PFAL in Japan. During the first 2 weeks of the experiment, 300 plants for each treatment were grown in a urethane cube mat at a density of 1847 plants per m^2^ (Supplementary Data Fig. S2). After 2 weeks, 200 healthy plants were randomly selected and transplanted into a custom-built nutrient film technique (NFT) system for weeks 3 and 4 at a density of 314 plants per m^2^ (Supplementary Data Fig. S2). The NFT system used the same reverse osmosis water, hydroponic fertilizer and concentration. After 4 weeks, 60 healthy plants were randomly selected and transplanted into a similar NFT system for weeks 5 and 6 but at a density of 63 plants per m^2^ (Supplementary Data Fig. S2).

### Plant growth analysis

Each experimental run began with 300 seeds planted per treatment. Every week for a total of 6 weeks, five to ten plants were randomly selected and harvested for destructive measurements. To minimize edge effects, only plants surrounded by neighbouring plants in the most evenly distributed light area were used for data collection (Supplementary Data Fig. S1). Border and corner plants were excluded due to increased exposure to possible airflow and microclimatic variability, which can alter growth and make them less representative of the overall treatment. Plants were measured for total shoot fresh mass and leaf area, and finally dried for 1 week at 80 °C in a constant-temperature oven for dry shoot mass measurement. We determined leaf surface area values using an LI-3100C Area Meter (LI-COR Biosciences, Lincoln, NE, USA), and all shoot biomass was used to measure average leaf surface area and specific leaf area. Every effort was made to flatten leaf tissue as much as possible during leaf surface area measurements.

For the plant shape analysis, plants were grown under the same experimental conditions. The main axis, a plant height parameter (taken from side images) and canopy equivalent diameter were measured (Fig. 5B).

Once a week, five randomly selected and consistently tagged plants were temporarily removed, and side- and top-view images were captured with plants placed inside a 64 × 64 × 64 cm white photography box illuminated with white LED light. Images were captured using an iPhone 12 Pro Max (Apple Inc., Cupertino, CA, USA) at 1× (no zoom), with the camera focused on the lettuce plant and positioned at a fixed distance of ∼70 cm from the centre of the urethane propagation cube where the seed was inserted. Plants were imaged without any structural support; therefore, the recorded plant height reflects the natural height under gravitational constraints, not the maximum extended length. From the side images, plant height (main axis length) was measured, while top-view images were used to calculate canopy equivalent diameter. Plant shape characteristics, which include top surface area, side surface area, canopy coverage (also referred to as ‘extent’ in MathWorks, 2024), main axis length and canopy equivalent diameter were analysed using EasyPCC V2 software (Guo *et al*., 2017), which applies a decision-tree-based segmentation model to accurately extract vegetation features from images under varied lighting conditions.

The canopy equivalent diameter of the circle (Fig. 5A) is of the same area as the pixel surface area of vegetative tissue from the top canopy using the following equation: $\sqrt{4\times top\;surface\;area÷\pi }$ (MathWorks, 2024). Canopy coverage was measured as the ratio of the vegetative pixel area (from the top-view image) to the total area of the bounding box, which includes both plant and background space (Fig. 5A). Top and side surface area were also measured (Fig. 5A, B). To the greatest extent possible, plants were carefully removed and promptly returned to the hydroponic system to minimize any adverse effects on plant growth.

### Photosynthesis analysis

The steady-state CO_2_ assimilation rates (*A*net) and stomatal conductance (*g*_s_) of all treatments were measured in fully expanded young leaves using a portable photosynthesis system (LI-6400XT, LI-COR Biosciences, Lincoln, NE, USA) with a clear top chamber, following the methods of Qu *et al*. (2021) and Yoshiyama *et al*. (2024), within their respective treatment environments using the same 5000-K W lights and FR lights as during plant growth. The CO_2_ reference concentration (CO_2_R) was set and controlled at 400 µmol mol^−1^, while CO_2_S corresponds to the measured sample CO_2_ concentration at the outlet of the chamber. Thus, all reported values were obtained under a controlled and stable CO_2_R of 400 µmol mol^−1^. Four weeks into the experiment, Anet and *g*_s_ were measured in five randomly selected plants grown under W light. Regarding light conditions, 300 µmol m^−2^ s^−1^ represented the light intensity and was also used during gas exchange measurements. Measurements were conducted under the growth LEDs, and the LED output was confirmed prior to each measurement session to ensure consistency with the growth light environment. In addition, the LA-105 Light Analyzer was used at the leaf surface immediately before measurement to verify that incident PPFD at the sampled area was 300 µmol m^−2^ s^−1^, accounting for canopy height and plant growth over time. Therefore, the reported light intensity corresponds to the actual irradiance experienced by the measured leaf. Measurements were first taken under 300 μmol m^−2^ s^−1^ of W light for 5 min. Far-red light was then added, and once *A* and *g*_s_ stabilized, they were recorded again under W + FR light for 5 min.

The same procedure was applied to five randomly selected plants grown under W + FR light at the 4-week stage: initial measurements were taken under 300 μmol m^−2^ s^−1^ of W light for 5 min, then FR light was supplemented. Once Anet and *g*_s_ stabilized, they were recorded again under W + FR light for 5 min.

Leaf absorptance (*α*) was measured using a custom-made integrating sphere, based on the equation *α* = 1 − (*T* + *R*), where *T* is transmittance and *R* is reflectance. In this integrating sphere, light from a tungsten lamp was guided through an optical fibre and converted into parallel rays using a convex lens before being directed onto the sample. The intensity and spectrum of transmitted and reflected light were measured with a JAZ spectrometer (Ocean Optics, Orlando, FL USA). For accurate determination of absolute reflectance, a 100 % reflectance standard plate was used. To minimize the influence of reflected light from the sample holder when measuring leaf reflectance, a custom holder was fabricated using a 3-D printer and coated with a commercial matte black paint known to have near 100 % light absorptivity.

Photosynthetic gas exchange measurements for 100 s under conditions without FR light followed by a 100-s measurement with FR illumination were also conducted (Supplementary Data Fig. S4).

### Ascorbic acid and pigment analysis

For lettuce quality analysis at the end of week 5, the contents of ascorbic acid (vitamin C), total chlorophyll *a + b*, total carotenoids, and anthocyanin were measured with four to ten plants measured per treatment (Levine *et al*., 2023; Hayashi *et al*., 2024). We selected week 5 for measurements to ensure consistent sampling and reproducibility, as plants at week 6 risked becoming impractically large and less representative of typical consumption stages across repeated trials. For ascorbic acid analysis, two fully expanded, healthy mature leaves were immediately harvested and thoroughly ground with a pestle and mortar. The juice was pipetted into a 2-mL vial using water for sample dilution. The ascorbic acid in each treatment was quantified by using Reflectoquant^®^ Ascorbic Acid Test strips (Sigma–Aldrich Canada Co., Ontario, Canada) together with an RQ Flex^®^ plus reflectometer instrument reader (Merck Darmstadt, Germany) (Furuta *et al*., 2025).

For anthocyanin, chlorophyll and carotenoid analysis, four 6.4-mm diameter (32-mm^2^ holes) discs were punched on large, fully expanded leaf blades in the darkest region areas that avoided the midrib. We carefully addressed potential anthocyanin variability by collecting all leaf discs from the upper ∼30 % portion of the outer edge region of leaves, where anthocyanins most prominently accumulated. Within this region, samples were randomly collected to avoid positional bias and to ensure representative sampling. The mass of the four leaf discs was immediately weighed to also assess pigment, anthocyanin and ascorbic acid concentrations on a leaf mass basis. For anthocyanin content index (ACI), 1.5 mL of a 50:45:5 volume of water, methanol and acetic acid was added according to Laby *et al*. (2000) to the four leaf discs and placed in a 2-mL vial with a homogenizer bead. This was subsequently shaken at 1100 rpm for 18 min in a bead-type crushing device (BMS-A20TP Shake Master, Biomedical Science Inc., Tokyo, Japan). The sample vial was subsequently centrifuged for 2 min and the supernatant was used for the analysis. Absorbances at 530.0 and 657.0 nm were measured using a spectrophotometer (UV-1280, Shimadzu Corporation, Kyoto, Japan), and the relative values of anthocyanin content were determined (Laby *et al*., 2000).

For chlorophyll and carotenoid analysis, 1.5 mL of 80 % acetone was added to the four leaf discs and the preparation was placed in a 2-mL vial along with a homogenizer bead. This was subsequently shaken at 1100 rpm for 6 min in the same crushing device. The sample vial was subsequently centrifuged for 2 min. Absorbances at 750.0, 663.2, 646.8 and 470.0 nm were measured and the chlorophyll and carotenoid contents were determined using equations from Lichtenthaler (1987).

### Gene expression analysis

For anthocyanin biosynthetic gene expression analysis, leaves of 5-week-old plants were sampled when lights had been on for 15 h of their 16-h photoperiod. Anthocyanin biosynthetic genes, including *CHS*, *F3H*, *DFR*, *ANS* and *UFGT*, were analysed (Supplementary Data Table S1). Total RNA was extracted using an RNeasy Plant Mini Kit (Qiagen N.V., Hilden, Germany) and reverse-transcribed using a PrimeScript™ RT Reagent Kit with gDNA Eraser (Takara Bio, Japan). RT–qPCR assay was performed with TB Green^®^ Premix Ex Taq™ II (Takara Bio, Japan) using a Step One Plus Real-Time PCR System (Thermo Fisher Scientific, USA). Eight biological replicates were applied for each gene. *Actin* (ACT, AB359898) was used as an internal control to normalize different samples. The primer sequences used in this analysis are listed in Supplementary Data Table S1.

### Statistical analysis

The experiment followed a randomized complete block design, with five to ten experimental units for each of the four treatments. Plant numbers per harvest varied due to scheduled changes in planting density. In weeks 1 and 2 we propagated 300 plants, allowing >20 viable samples, although we limited sampling to 10 plants. In weeks 3 and 4, with 100 plants per treatment we maintained a sample size of 10. By weeks 5 and 6, density dropped to 30 plants per treatment, limiting sampling to 5–7 plants while avoiding non-represented plants on the edges. The same number of replicates was always included between the two light conditions at the respective week stages. We conducted the experiment as four independent experimental runs over time using the same PFAL facility, lighting treatments, and cultivation protocols. For each run, measurements were first summarized at the treatment level, and the resulting values were then combined across runs for statistical analysis. The data were analysed using JMP Pro 15 software, developed by the JMP business unit of the SAS Institute, using the Student t-test and Tukey–Kramer honestly significant difference (HSD) test at *P* = 0.05 to determine significant differences among measured parameters based on light treatment.

## RESULTS

### Plant growth

**Fig. 2.**
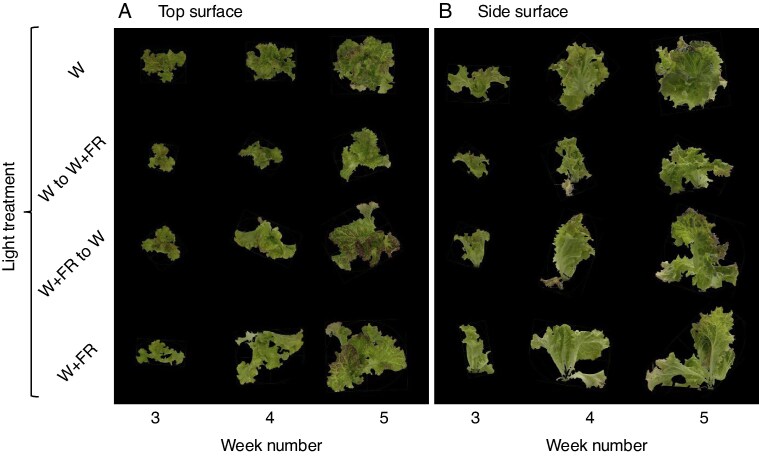
(A) Top surface and (B) side surface views of representative red leaf lettuce grown under continuous white light (W), W for 4 weeks followed by W plus far-red (W + FR), W + FR followed by W, and continuous (W + FR) measured 3, 4 and 5 weeks after respective treatments. The images depict the morphological differences between the four lighting treatments, highlighting the increased leaf expansion and more robust canopy development in the W + FR treatment compared with the W treatment.

The W + FR treatment significantly increased the top canopy surface area (Fig. 5C, D), side canopy surface area (Fig. 5E, F) and canopy equivalent diameter (Fig. 5K, L) at weeks 3, 4 and 5 compared with the W and W to W + FR treatments. These increases in top canopy surface area and canopy equivalent diameter are also visually apparent in the representative images in Fig. 2A, while changes in side canopy surface area can be observed in the representative images in Fig. 2B. At week 3, the W treatment exhibited greater canopy coverage compared with the W + FR treatment; however, by weeks 4 and 5 canopy coverage was similar across all treatments.

**Fig. 3.**
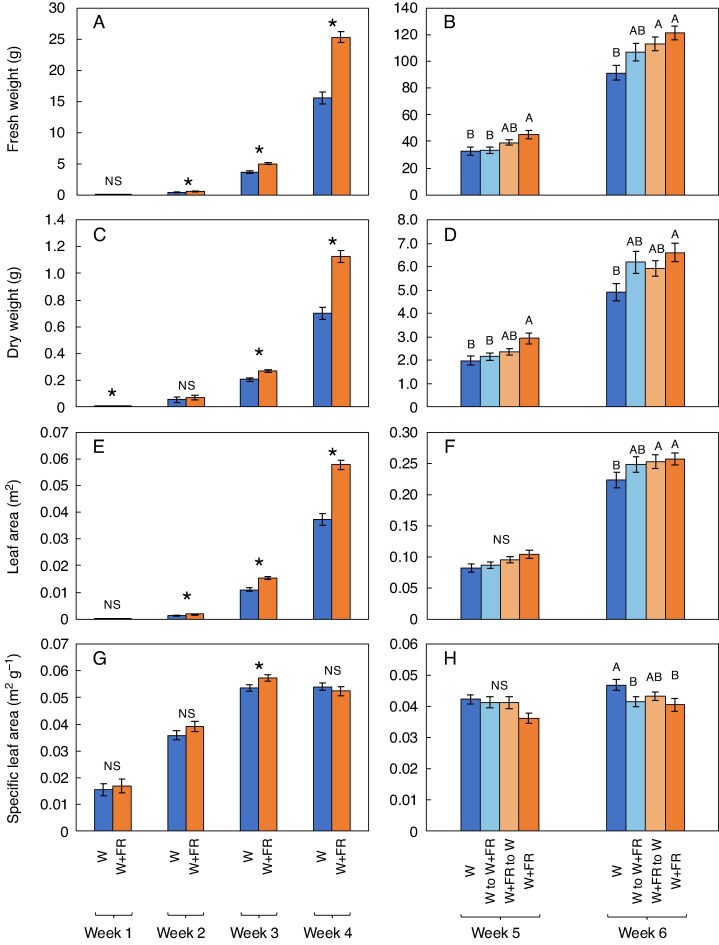
(A) Fresh weight of red leaf lettuce shoots under different lighting treatments from week 1 to week 4. (B) Fresh weight of red leaf lettuce shoots under different lighting treatments from weeks 5 and 6. (C) Dry weight of red leaf lettuce shoots under different lighting treatments from weeks 1 to 4. (D) Dry weight of red leaf lettuce shoots under different lighting treatments from weeks 5 and 6. (E) Leaf area of red leaf lettuce under different lighting treatments from weeks 1 to 4. (F) Leaf area of red leaf lettuce under different lighting treatments from weeks 5 and 6. (G) Mean specific leaf area of red leaf lettuce shoot from week 1 to 4. (H) Mean specific leaf area from week 5 and 6. Bars represent standard errors (*n* = 5–10). *Indicates significant differences between two treatments, as determined by Student's t-test (*P* ≤ 0.05); Different capital letters indicate statistically significant differences between multiple treatments within each time point, as determined by Tukey’s HSD test (*P* ≤ 0.05). NS, not significant.

Regarding destructive harvest parameters, W + FR resulted in increased shoot fresh and dry weight across most weeks relative to W treatments (Fig. 3A–D). In week 1, the W + FR treatment resulted in greater dry weight (4.5 ± 0.3 mg) compared with the W treatment (3.5 ± 0.2 mg) but not statistically different fresh weight (Fig. 3A, C). In week 2, The W + FR treatment had significantly higher fresh weight (599.5 ± 15.3 mg) but similar dry weight relative to the control (fresh weight: 468.5 ± 16.4 mg) (Fig. 3A, C). The differences became more pronounced starting from week 3 with fresh and dry weights (Fig. 3A–D). As the experiment progressed, plants under the W + FR treatment consistently exhibited the highest dry and fresh weights, while those under the control (W) produced the lowest (Fig. 3A–D). Interestingly, the W + FR to W treatment had a similarly high fresh weight (113.2 ± 5.2 g) to the W + FR treatment (121.3 ± 5.2 g) by week 6 (Fig. 3B). The treatments where plants were switched from W to W + FR (6.2 ± 0.5 g) or from W + FR to W (5.9 ± 0.3 g) yielded intermediate dry weight by week 6 (Fig. 3D). Although the growth-promoting effects of FR light have been reported, few studies have assessed these effects under commercially relevant production conditions. Here we conducted an experiment using the facility, nutrient management system and cultivation equipment of a commercial PFAL company in Japan currently operating four active facilities. Plants were initially grown at 1847 plants per m^2^ and transplanted to 314 and 63 plants per m^2^ following standard commercial density reduction workflows. This dynamic adjustment mirrors real-world practices to optimize production. Our findings provide new insights into how FR supplementation affects lettuce biomass and morphology across different density stages as well as anthocyanin biosynthetic gene expression, quality metrics and photosynthetic parameters. Statistical analysis confirmed that these differences were significant, as indicated by the distinct groupings identified by the Tukey–Kramer HSD test (Figs 2 and 3).

Regarding leaf area, the W + FR treatment generally promoted greater leaf expansion compared with the control (W), particularly in the early weeks (weeks 2–4), with the most notable difference observed in week 4 (Fig. 3E). As the plants matured from week 5 to week 6, leaf area across all treatments became similar (Fig. 3F). Regarding specific leaf area (SLA), all treatments were similar across all weeks except week 3, in which W + FR treatment had significantly increased SLA relative to the control. These results indicate that FR supplementation initially enhances leaf area and SLA (in week 3), but these effects become less pronounced as the lettuce plants reach full maturity. The Tukey–Kramer HSD test supported these observations, confirming the significant differences (Fig. 3).

### Photosynthetic parameters

**Fig. 4.**
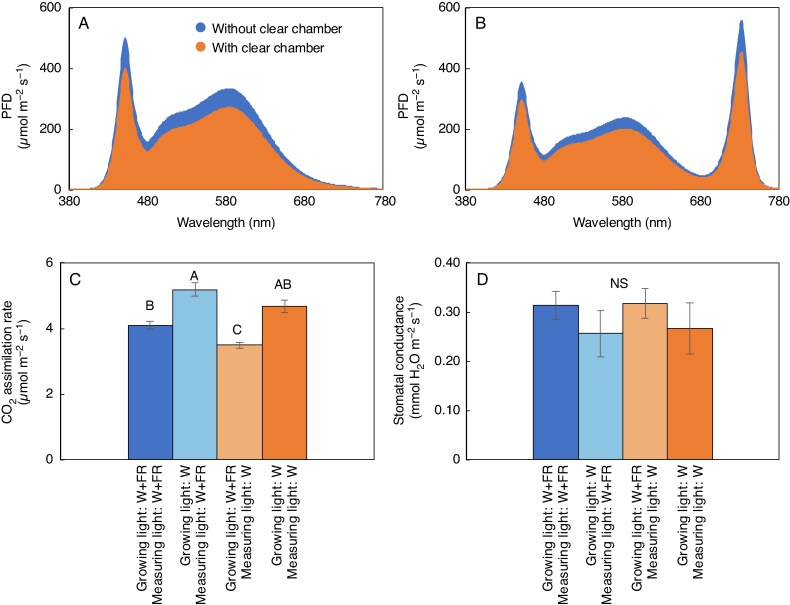
Growth light spectra with and without the clear top chamber. (A) White light (W) and (B) white plus far-red light (W + FR). (C, D) Red leaf lettuce measurements taken 4 weeks after seeding of treatments 1–4 of (C) CO_2_ assimilation rate and (D) stomatal conductance. Bars are standard errors (*n* = 4 or 5). Different capital letters indicate statistically significant differences between multiple treatments within each time point, as determined by Tukey’s HSD test (*P* ≤ 0.05). NS, not significant.

Regarding CO_2_ assimilation rates, leaves acclimated to W and measured under W + FR had the highest CO_2_ assimilation rates, significantly exceeding that of FR-acclimated leaves measured under either W or W + FR (Fig. 4C). In both the W treatment and the treatment where plants were switched from W + FR to W, CO_2_ assimilation rates were enhanced when FR light was supplemented during measurement compared with when it was not provided. There was no statistically significant difference among the stomatal conductance values among all treatments (Fig. 4D).

Regarding emitted light and leaf absorptance, FR light (700–750 nm) was absorbed substantially less than photosynthetically active radiation (PAR; 400–700 nm) photons in both red and green leaves (Table 2 and Supplementary Data Fig. S3). While absorption within the 400–700 nm range exceeded 64 % in red leaves and 53 % in green leaves, total FR absorption was only 7.6 and 6.3 %, respectively. Notably, the longer FR waveband (715–750 nm) had the lowest absorption, with red leaves absorbing just 4.0 % and green leaves 3.1 %. These results indicate a marked drop in absorptance beyond 715 nm. Thus, the photosynthetic contribution of FR photons, especially those above 715 nm, may be limited due to lower absorptance.

**Table 2.** Incident light and leaf absorptance (µmol m^−2^ s^−1^ and %) across the 400–750 nm spectrum in red and green leaves.

Colour	Waveband (nm)	Incident light (μmol m^−2^ s^−1^)	Red leaf absorptance (%)	Green leaf absorptance (%)
Blue	400–500	88.1	90.5	86.3
Green	500–600	136.9	75.4	51.6
Red	600–700	76.4	74.4	69.6
Far red	700–715	11.6	29.3	25.9
Far red	715–750	71.8	4.0	3.1
Total far red	700–750	83.4	7.6	6.3
Total photon flux density	400–750	385	64.0	53.3

Table 3 shows that adding FR light (700–750 nm) slightly increases total absorbed quanta beyond the 400–700 nm baseline, but the increase is modest, at only ∼2.7 % for red leaves and 2.6 % for green leaves. Under constant PFD substitution, overall absorption drops to ∼78 %, highlighting that FR is absorbed less efficiently than PAR. When absorbed quanta are matched (ideal conditions), FR inclusion is equal to total absorption compared with PAR alone.

**Table 3.** Absorbed quanta^1^ under different treatments.

	Emission	Red leaf absorptance	Green leaf absorptance
Present study^2^			
400–700 nm	100 % _PAR_ (normalized)	100.0 % (normalized)	100.0 % (normalized)
400–750 nm	100 % _PAR_ + 28 % _FR_	102.7 %	102.6 %
Fixed emission^3^		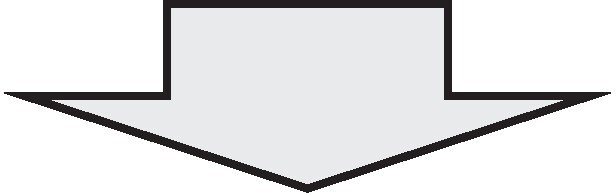	
400–700 nm	100 % _PAR_ (normalized)	100.0 % (normalized)	100.0 % (normalized)
400–750 nm	78 % _PAR_ + 22 % _FR_	78.7 %	78.8 %
Fixed absorption^4^			
400–700 nm	100 % _PAR_ (normalized)	100.0 % (normalized)	100.0 % (normalized)
400–750 nm	97 % _PAR_ + 28 % _FR_	100.0 %	100.0 %

^1^Proportion of incident photons absorbed by the leaf provided with extra FR compared with treatment in which only PAR photons were provided.

^2^Based on actual data as collected in our current study.

^3^Assuming total photon emission is fixed to the normalized standard, with the ratio of PAR against FR unchanged the absorption would be as indicated.

^4^Assuming leaf absorption is fixed to normalized standard, with the ratio of PAR against FR unchanged the emission should be as indicated.

Lastly, photosynthetic measurements under conditions without FR light followed by a 100-s measurement with FR illumination was conducted (Supplementary Data Fig. S4). Since the FR light contained a small amount of PPFD, a slight increase in the CO_2_ assimilation rate was observed; however, the effect was small. Therefore, under white-light conditions the enhancement of CO_2_ assimilation observed with FR illumination can be attributed mainly to the contribution of the FR component itself.

### Plant form: canopy architecture

**Fig. 5.**
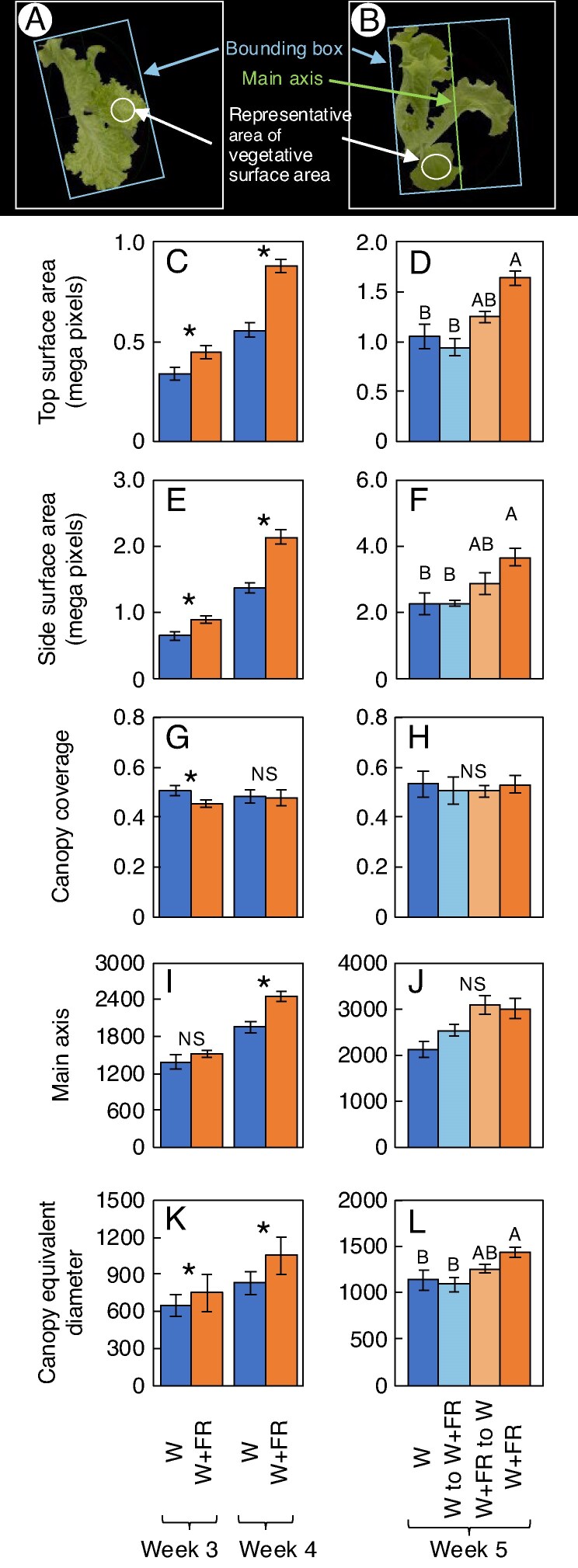
(A) Representative image of the top canopy and (B) representative side image of red leaf lettuce, with explanations of measured parameters. (C-H) in megapixels. (C) Top surface area from weeks 3 and 4. (D) Top surface area from week 5. (E) Side surface area from weeks 3 and 4. (F) Side surface area from week 5. (G) Canopy coverage (top canopy surface area, calculated as the actual number of vegetation pixels divided by the area of the bounding box) from weeks 3 and 4. (H) Canopy coverage from week 5. (I-L) in pixels. (I) Main axis from the side (a general reference of approximate plant height) from weeks 3 and 4. (J) Main axis from week 5. (K) Canopy equivalent diameter (diameter of the circle whose area is the same as the pixel surface area of vegetative tissue from the top canopy) from weeks 3 and 4. (L) Canopy equivalent diameter from week 5. Bars represent standard errors (*n* = 5). Capital letters indicate significant differences by Tukey’s HSD test (5 % level of significance). *Indicates significant differences between two treatments, as determined by Student's t-test (*P* ≤ 0.05). NS, not significant.

The impact of FR light on plant morphology was assessed by EasyPCC V2 software image analysis from top and side perspectives (Figs 2 and  5A, B). Regarding canopy surface area, the W + FR treatment consistently resulted in the largest top and side canopy surface area (top surface, 1.64 × 10^6^ ± 1.22 × 10^5^ pixels (px); side surface, 3.68 × 10^6^ ± 3.6 × 10^5^ px at 35 d) compared with the W treatment (top surface, 1.06 × 10^6^ ± 1.73 × 10^5^ px; side surface, 2.26 × 10^6^ ± 4.5 × 10^5^ px at 35 d) across each week, indicating that continuous FR exposure promotes significant lateral growth and canopy expansion (Fig. 5C–F).

Significant differences in canopy coverage, defined as the surface area of actual vegetation pixels divided by the bounding box area, were also found. On week 3 the W treatment had the highest canopy coverage, significantly outperforming the continuous W + FR treatment (Fig. 5G). However, as the experiment progressed the differences in canopy coverage between the treatments diminished, with all treatments showing similar canopy coverage in weeks 4 and 5 (Fig. 5G, H). This suggests that while initial differences in canopy coverage may exist, the plants’ overall coverage tends to equalize over time.

The main axis, an indicator of plant height, also varied significantly across the different treatments (Fig. 5I). By week 4, the W + FR treatment had significantly increased height relative to the W treatments (Fig. 5I). However, in weeks 3 and 5 there were no significant differences in the main axis across all light treatments (Fig. 5I, J).

Regarding the canopy equivalent diameter, which is the diameter of a circle with an area equivalent to the contour area of the canopy, the W + FR treatment consistently resulted in the largest diameters in all weeks (Fig. 5K, L). This trend was evident from the earliest measurements in week 3, when W + FR significantly outperformed the W treatment. Over time, the W + FR treatment maintained its dominance, resulting in the largest canopy diameters by the final measurement in week 5 (Fig. 5L). The W treatment, on the other hand, resulted in the smallest diameters, indicating that white light alone does not promote the same level of canopy expansion growth as FR-supplemented treatments (Fig. 5K, L). The intermediate treatment W to W + FR showed canopy equivalent diameters statistically smaller than W + FR treatment by week 5 and the W + FR to W treatment showed canopy equivalent diameters between the W and W + FR treatments by week 5 (Fig. 5L).

### Photochemical and gene expression analysis

**Fig. 6.**
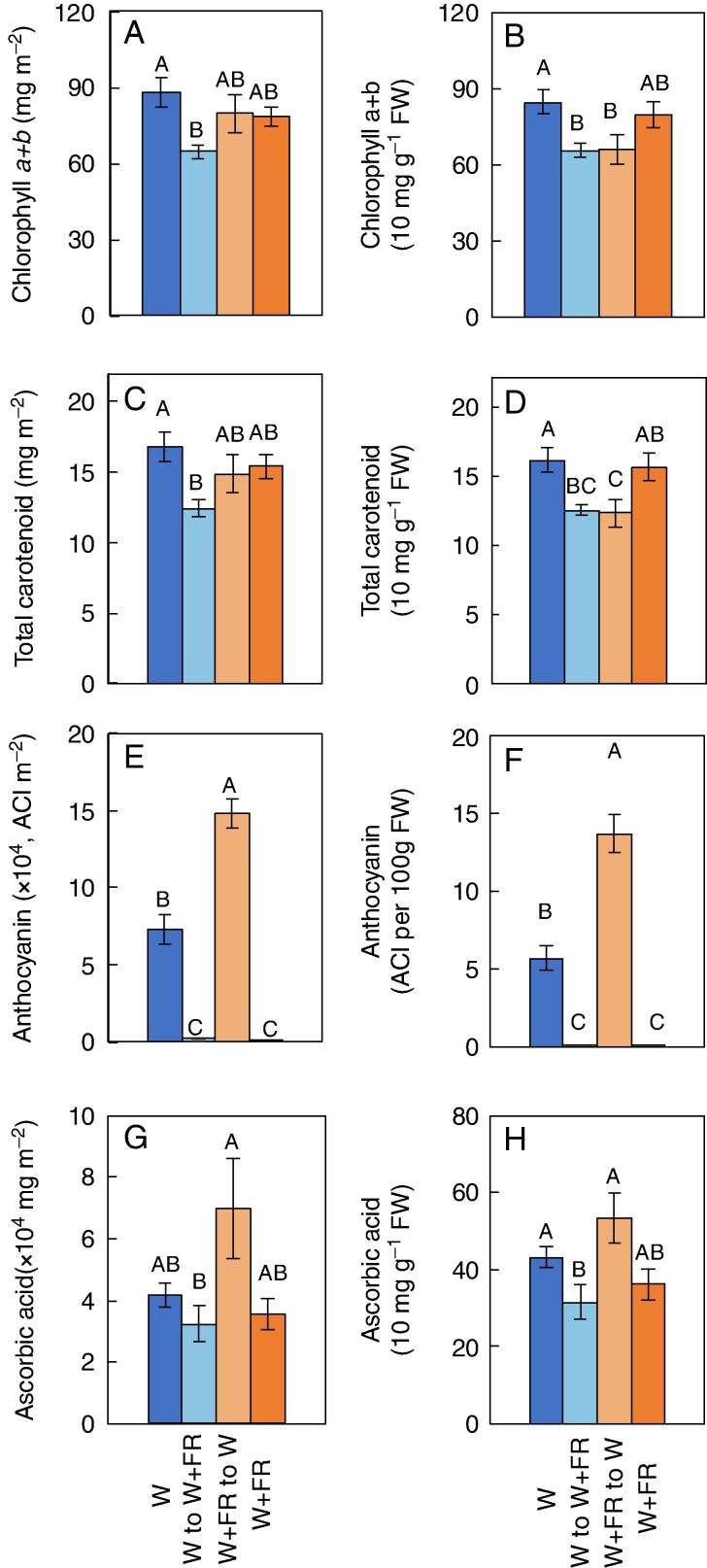
(A) Red leaf lettuce total chlorophyll a + b concentrations 5 weeks after seeding with all treatments per m² and (B) concentration per g FW. (C) Lettuce total carotenoid concentrations 5 weeks after seeding with all treatments per m² and (D) total carotenoid concentration per g FW. (E) Anthocyanin content index (ACI) in red leaf lettuce 5 weeks after seeding with all treatments per m². (F) ACI per g FW. (G) Ascorbic acid concentration 5 weeks after seeding with all treatments per m². (H) Ascorbic acid concentration per g FW. Bars are standard errors (n = 4–10). Different letters indicate statistically significant differences between treatments, as determined by Tukey's HSD test (P ≤ 0.05).

Regarding chlorophyll *a* + *b* concentration, the control (W) consistently resulted in the greatest chlorophyll content, suggesting that this particular 5000-K W spectral treatment alone is optimal for maximum chlorophyll synthesis and accumulation relative to the other lighting treatments in this study (Fig. 6A, B). The W to W + FR treatment had the lowest chlorophyll levels on both per unit area and per unit weight bases, indicating that introducing FR later in the growth cycle might reduce chlorophyll content. The W + FR to W treatment also had significantly lower chlorophyll per unit weight (Fig. 6B).

Ascorbic acid content was also measured to assess the nutritional quality. The control treatment W and W + FR to W treatments had the highest ascorbic acid contents, indicating that W light alone or when applied at the later stage is most conducive to maintaining or enhancing this antioxidant in lettuce leaves (Fig. 6G, H). In contrast, the W to W + FR treatment resulted in the lowest ascorbic acid content, suggesting that FR supplementation, particularly when applied later in the growth cycle, may diminish the accumulation of ascorbic acid (Fig. 6G, H). The W + FR treatment showed intermediate ascorbic acid concentrations, indicating that the timing of FR exposure plays a critical role in influencing this nutrient’s concentration (Fig. 6G, H).

**Fig. 7.**
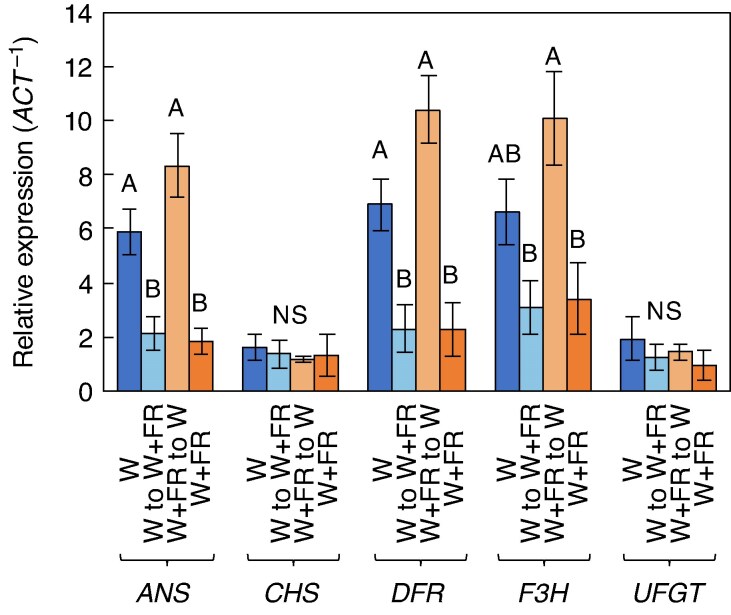
Anthocyanin biosynthetic gene expression analysis of fresh leaf tissue from red leaf lettuce 5 weeks after seeding under different lighting treatments: white light (W), white light followed by far-red (W to W + FR), far-red followed by white light (W + FR to W) and continuous far-red (W + FR). Capital letters indicate statistically significant differences between treatments, as determined by Tukey’s HSD test (*P* ≤ 0.05). Bars represent standard errors (*n* = 10). ifferent letters indicate statistically significant differences between treatments, as determined by Tukey’s HSD test (*P* ≤ 0.05).

The relative expression levels normalized to actin (/ACT) of five key anthocyanin biosynthetic genes – *ANS*, *CHS*, *DFR*, *F3H* and *UFGT –* were also measured (Fig. 7). Analysis of the data suggests that the W and the W + FR to W treatments exhibited significantly higher expression levels of *ANS*, *DFR* and *F3H* compared with the continuous W + FR treatment and W to W + FR treatments (Fig. 7). Since the gene expression analysis was measured at the week 5 stage, it is important to note that the W + FR to W treatment had already transitioned to the W treatment for 1 week and was no longer receiving FR supplementation at the time of measurement. In contrast, *CHS* and *UFGT* gene expressions showed no significant differences across any of the treatments, indicating that these genes were less responsive to the lighting conditions tested (Fig. 7).

### General evaluation

**Fig. 8.**
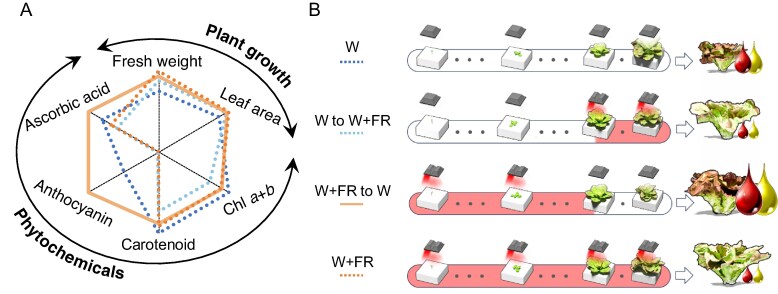
(A) Radar plot among four treatments and (B) summary illustration. The radar plot compares the effects on various growth and phytochemical parameters in plants of four different treatments: white light (W), white light followed by far-red (W to W + FR), far-red followed by white light (W + FR to W) and continuous far-red (W + FR). The parameters measured are fresh weight, leaf area, chlorophyll a + b content, total carotenoid content, anthocyanin content and ascorbic acid content. These parameters are categorized into two broad groups: plant growth (fresh weight and leaf area) and phytochemicals (chlorophyll, carotenoids, anthocyanin and ascorbic acid). The illustration of summary visually shows the general timeline for each treatment and the light regime transitions, showing how the type and timing of light exposure influenced lettuce growth and phytochemical accumulation.

Lastly, to summarize all the results, the radar plot illustrates the comparative effects of all treatments on key plant growth and phytochemical parameters, including fresh weight, leaf area and chlorophyll *a* + *b*, total carotenoid, anthocyanin and ascorbic acid concentrations (Fig. 8). The W + FR to W treatment generally demonstrates superior outcomes across all parameters while the W + FR treatment leans more towards superior plant growth parameters and compromises on anthocyanin phytochemicals relative to other treatments. The W to W + FR treatment shows a stronger tendency for plant growth parameters over phytochemical synthesis. Lastly, the W treatment leans more towards phytochemical synthesis while sacrificing plant growth (mainly fresh weight) parameters. This analysis underscores the differential impact of dynamic light conditions on both growth and phytochemical profiles.

## DISCUSSION

The present work showed that the FR treatment led to an increase in leaf area in lettuce (Fig. 3E, F), improving light interception and significantly increasing biomass production (Fig. 3A–D). However, this was accompanied by a reduction in the localized concentration of anthocyanins (Fig. 6E, F). Furthermore, compared with the W treatment, the W to W + FR treatment resulted in a significant decrease in total carotenoid concentrations on both area and weight bases (Fig. 6C, D). However, while total carotenoids per leaf area were significantly lower following the W to W + FR treatment, carotenoids per leaf mass were not significantly different from the W treatment (Fig. 6C, D). Thus, there is a potential trade-off between yield and quality.

Regarding the higher SLA observed in the W + FR treatment at week 3 compared with the W treatment, this response is likely related to plant developmental stage and canopy structure. At week 3, plants were relatively small and grown at a low density of 314 plants per m^2^, which likely allowed FR photons to reach a larger proportion of the leaf tissue. Under these conditions, FR exposure may have promoted leaf expansion relative to biomass accumulation, resulting in higher SLA. By week 4, plants were larger and canopies more developed, increasing mutual shading and reducing the proportion of FR photons reaching individual leaves. This change in canopy architecture likely diminished the relative effect of FR on leaf expansion, explaining why SLA differences between treatments were no longer observed across most other weeks.

Although the spectrophotometric measurements in Fig. 6E, F show significantly higher anthocyanin accumulation than those that appear in the representative images in Fig. 2, it is important to note that anthocyanin is highly localized. The sampled leaf discs were taken from the darkest regions with the highest pigmentation and do not reflect anthocyanin levels across the entire plant shown in Fig. 2. In contrast, the absence of FR light on week 5, 1 week prior to final harvest, increased the accumulation of desirable chemical compounds, including anthocyanins and ascorbic acid, in plants grown under W light 1 week before harvest (Fig. 6E–H). Thus, applying FR light only during the early growth phase could improve plant production, balancing nutritional quality and yield (Figs 3B and 6E–H).

Analysis of the radar (spider) plot data suggests that FR radiation applied during the initial two-thirds of the production cycle (weeks 0–4 of a 6-week cycle) can be strategically utilized to enhance plant growth and phytochemical content in plants grown under W light during the later stages in a commercial PFAL with this cultivar under these growing conditions (Fig. 8).

### Far-red light enhances plant growth by improving architecture and photosynthesis

The increased biomass accumulation observed with supplementary FR light (Fig. 3A–D), the increased CO_2_ assimilation rates with W-acclimated leaves measured under W + FR (Fig. 4C) and the expanded leaf canopy area (Fig. 5) suggest that FR supplementation enhances plant growth through a combination of increased canopy expansion and CO_2_ assimilation.

Several studies have shown that fluctuating light observed in natural light environments causes photosystem 1 (PSI) photoinhibition, highlighting the potential vulnerability of PSI under changing light conditions (Suorsa *et al*., 2012; Kono and Terashima, 2014; Yamori *et al.*, 2015; Yamori and Shikanai, 2016; Yamori *et al*., 2016*a*, *b*). The addition of FR to fluctuating light suppressed PSI photoinhibition via keeping oxidation of P700, the reaction centre of PSI (Kono *et al*., 2017). More recently, it has been reported that FR exerts beneficial effects on photosynthesis in fluctuating light by exciting PSI and accelerating the relaxation of non-photochemical quenching (NPQ) in the photosystem 2 (PSII) antenna system, resulting in increases in photosynthesis (Kono *et al*., 2020). At the mechanistic level, FR illumination primarily drives excitation of PSI, resulting in the oxidation of P700 and stimulation of cyclic electron flow of PSI (CEF-PSI). This process reinforces ΔpH formation and promotes adenosine triphosphate (ATP) synthesis (Kramer *et al*., 2004; Yamori and Shikanai, 2016; Shikanai, 2024).

Furthermore, FR excitation facilitates quicker relaxation of NPQ and PSII centres. This improves the effective quantum efficiency of PSII (ΦPSII) and CO_2_ fixation. Meanwhile, PSI is alleviated by over-reduction under dynamic light conditions (Yamori and Shikanai, 2016; Kono *et al*., 2020; Shikanai, 2024; Lazzarin *et al*., 2025). The magnitude of these responses is largely determined by the spectral composition, light intensity and physiological state of the leaf (Evans, 1987; Chow *et al*., 1990; Taylor *et al*., 2019). Despite these advances, understanding the underlying mechanisms of FR action remains crucial to the clarification of how FR light modulates and safeguards photosynthesis across variable light environments.

On the contrary, Zhen and Bugbee (2020) reported that substitution of FR photons maintained an equivalent canopy quantum yield, thereby suggesting that FR photons can contribute as effectively as PAR photons to photosynthesis when acting synergistically. However, more recently Jeong *et al*. (2024) suggested that under FR light substitution plant growth may become more dependent on total photon capture rather than photosynthetic efficiency. Jeong *et al*. (2024) reported a significant reduction in the net photosynthetic rate per unit leaf area when a 20 % substitution of FR photons was applied to FR-acclimated leaves. In a recent review, Shomali *et al*. (2025) stated ‘If such a link exists between the chloroplast and the nucleus, then it raises the opinion that FR perception takes part in driving photosynthesis through the perception of chloroplastic retrograde signals’. This perspective emphasizes that if FR is shown to regulate photosynthesis beyond simple photochemical responses, then its integration into light management strategies could be more foundational than currently understood. Amelii *et al*. (2026) also discusses FR-driven photosynthesis in eukaryotes and its potential in future bioengineering to increase light absorption. However, such a regulatory mechanism between FR perception and photosynthetic enhancement remains to be fully elucidated. Until such a mechanism is conclusively demonstrated, the proposal to regard photons in the 400–750 nm range as equivalent photosynthetic photons, without accounting for leaf absorptance cannot be considered best practice.

It is also noteworthy that independent non-profit organizations such as Design Lights Consortium (DLC), in its latest Technical Requirements for LED-Based Horticultural Lighting, Version 3.0, maintains its evaluation of photosynthetic photon flux (PPF) and photosynthetic photon efficacy (PPE) within the American National Standards Institute (ANSI)/Illuminating Engineering Society (IES) LM-79 standard of measuring photons in the 400–700 nm range. This standard reflects the well-established definition of PAR as the wavelengths most directly contributing to photosynthesis.

Overall, FR photons fall outside the established PAR spectrum (400–700 nm) and contribute less to the energy that directly drives photosynthetic reactions, resulting in a small effect in a stable, steady-state light environment (Fig. 4). Today, commercial CEA typically relies on stable, high-PAR lighting environments, focusing primarily on achieving photosynthetic photon flux density (PPFD) and daily light integral (DLI) targets. Nevertheless, with the advent of LEDs and multichannel lighting systems, horticultural lighting technologies are advancing towards more dynamic light management, and insights into PSI stability under varying light conditions may become increasingly relevant to CEA.

Far-red light influences not only photosynthesis (Fig. 4C) but also plant morphology (Figs 2, 5C, D, K, L and 8), exerting significant effects on both physiological and structural aspects of plant growth. The absorption of FR photons influences phytochrome systems rather than chlorophyll, meaning the plant’s response is more focused on structural changes for optimizing light use efficiency rather than boosting photosynthetic output. The present work showed that the supplementary FR treatment led to enlarged leaves (Fig. 2) and expanded canopy size (Fig. 5C, D), which enhanced light interception and, together with increased CO_2_ assimilation (Fig. 4C), likely contributed to the significant increase in plant biomass (Fig. 3A–D). These results indicate that FR promotes plant growth through both improved photosynthetic performance and morphological changes. These findings are consistent with previous research that showed FR supplementation and substitution promotes stem elongation and leaf expansion (Zhen *et al*., 2020; Eylands and Mattson, 2023; Kusuma *et al*., 2023), which is a response likely to enhance light-foraging capacity (Franklin, 2008), and cell wall-modifying mechanisms are vital regulatory points for control of this elongation response (Sasidharan *et al*., 2008).

In conclusion, supplementary FR light effectively promoted plant growth by stimulating both morphological changes and photosynthetic activity. W + FR-acclimated leaves showed increased CO_2_ assimilation rates when measured under W + FR light, although the rates were not as high as those of W-acclimated leaves measured under the same light condition. These results highlight that FR supplementation contributes to enhanced biomass production through its combined effects on plant architecture, light interception and, to some extent, CO_2_ assimilation under the tested air temperatures and light intensities.

Such architectural changes may be particularly advantageous under high-density PFAL planting conditions, where maximizing light interception per unit ground area is essential for optimizing productivity. Far-red LEDs are also among the most efficient in terms of photon output per unit energy input (µmol J^−1^) due to the lower energy of FR photons, making them valuable for reducing energy consumption in PFAL systems (Kusuma *et al*., 2020).

### Early-stage FR radiation promotes growth without compromising the final concentrations of functional compounds

While continuous FR supplementation throughout the entire 6-week growth period enhances plant biomass (Fig. 3B, D), it results in a decrease in the concentration of phytochemicals, such as anthocyanin and ascorbic acid (Fig. 6E–H). In contrast, this study revealed that supplementing FR radiation (W + FR) only during the first 4 weeks of growth and following this with W results in final plant growth comparable to that observed with continuous FR radiation. W + FR to W also significantly increased anthocyanin concentrations (Fig. 6E, F) and maintained greater ascorbic acid concentration relative to W to W + FR and similar ascorbic acid concentrations to W and W + FR treatments (Fig. 6G, H). This suggests that the initial exposure to FR followed by a shift to W light may trigger a stress response or a metabolic shift that leads to increased anthocyanin accumulation. Moreover, since the W + FR to W plants were taller and closer to the W lights at the time of measurement, the increased light intensity they received could have further amplified anthocyanin synthesis, explaining the significantly greater concentration compared with the continuous W treatment.

The gene expression analysis revealed that W + FR to W treatment resulted in significantly higher expression levels of key anthocyanin biosynthetic genes (*ANS*, *DFR* and *F3H*) compared with the continuous W + FR treatment and the W to W + FR treatment (Fig. 7). Some of these anthocyanin biosynthesis genes expressed in our study are also consistent with ones expressed in Goto *et al*. (2016). Since the W + FR to W plants were generally taller and closer to the lights, thereby receiving greater light intensity than the W treatment, which may explain why their gene expression was numerically greater than in the W treatment. It is well known that anthocyanins contribute to photoprotection by attenuating excess light, while ascorbic acid mitigates oxidative stress by scavenging reactive oxygen species under high-light conditions (Szymańska *et al*., 2017). While continuous W + FR treatment alone might reduce *ANS*, *DFR* and *F3H* gene expression, it may still play a crucial role in enhancing gene expression when followed by only W light. Alternatively, FR radiation induced a shade avoidance response in the plants, leading to thinner leaves and a shade-type phenotype as a low-light response. Consequently, exposure to W light later could trigger a stress response and thus the plants may have exhibited an increased sensitivity to moderate light (300 μmol m^−2^ s^−1^), which is not considered low for PFAL lettuce production.

Our results specifically demonstrate that after 4 weeks of W + FR treatment, shifting to W light alone for one additional week is sufficient to significantly increase anthocyanin accumulation. This W + FR to W treatment maintains fresh mass comparable to continuous W + FR treatment while significantly enhancing anthocyanin pigmentation. It provides a practical light management strategy that growers can implement during the final production phase to stimulate leaf pigmentation through the plant’s natural stress response to spectral changes, without the need to invest in multichannel LED horticulture systems. Simply transplanting crops to a different section of a PFAL for increased spacing with a preset fixed spectrum of 5000 K white is sufficient to achieve this effect for ‘Red Fire’ red leaf lettuce.

These findings have important implications for the commercial production of red leaf lettuce, where leaf colour is often associated with perceived quality (Owen *et al*., 2015); however, while the W + FR to W treatment significantly increases anthocyanin levels relative to other light regimes, our study does not assess whether the absolute pigmentation achieved meets consumer expectations or commercial marketability thresholds. We therefore focus our conclusions on the physiological and production aspects of anthocyanin enhancement.

In addition to the W + FR to W treatment demonstrated in our experiment, other techniques have been reported to stimulate anthocyanin production. These include but are not limited to end-of-production root-zone temperature control (Levine *et al*., 2023; Hayashi *et al*., 2024), nutrient stress (Jezek *et al*., 2023), supplementary blue (B) light application (Kelly and Runkle, 2023; Zhu *et al*., 2024*a*, *b*), controlled exposure to 310 nm UV-B (Goto *et al*., 2016) and 315–399 nm exposure to UV-A (Kelly and Runkle, 2023). Each of these approaches applies a different abiotic stressor to trigger secondary metabolite production, including anthocyanins, in leafy greens.

However, not all commercial PFALs, such as the commercial PFAL used in our study, are equipped with multichannel lighting systems with independently dimmable channels such as UV-A, UV-B, B and W, root-zone temperature control systems or targeted individual macronutrient management capabilities. Consequently, the range of techniques commercially available for controlling anthocyanin accumulation may be constrained by the technological infrastructure of each PFAL.

Although many previous works report that increased FR supplementation decreased phytochemical concentrations, including anthocyanin, carotenoid and ascorbic acid in lettuce (Li and Kubota, 2009; Stutte *et al*., 2009; Zou *et al*., 2023), our study showed that early-stage FR radiation promotes growth without affecting the final levels of functional compounds. Understanding the optimal balance between FR exposure and pigment accumulation is essential for producing visually appealing lettuce with high anthocyanin content, particularly in CEA systems, where light quality can be precisely managed.

### Practical implications

One of the significant findings of this study is the demonstrated effect of FR supplementation under commercially realistic high-density growing environments, where spectral quality, planting density and the resulting canopy light interception collectively influence shoot biomass and pigment accumulation. By performing week-by-week destructive harvests across three practical spacing densities representative of commercial Japanese PFAL setups, this study provides a uniquely detailed dataset capturing changes in shoot biomass, leaf area and SLA. These results not only validate the practical utility of the W + FR to W light shift for enhancing anthocyanin accumulation without compromising fresh mass but also offer concrete reference data for modelling potential head lettuce yields in NFT-based PFAL systems.

Furthermore, the promotion of lateral growth and canopy expansion under FR light, as evidenced by the increased canopy surface area and equivalent diameter (Fig. 5A–D, K, L), suggests that plants grown under FR exposure may require more space to avoid overcrowding and ensure optimal light penetration to lower leaves. The findings also indicate that FR exposure can influence canopy architecture in ways that affect planting density. For instance, plants that were exposed to FR light (W + FR) had significantly greater canopy surface area (Fig. 5C, D), top surface area, side surface area (Fig. 5E, F) and canopy equivalent diameter (Fig. 5K, L), relative to the control treatment. This suggests that they might need different planting densities for more efficient growth. Overall, FR exposure may require careful monitoring and potentially increased spacing to prevent excessive shading and competition among plants. The implications for commercial CEA operations are clear: while FR supplementation offers substantial benefits in terms of biomass accumulation (Fig. 3A–D), these must be balanced with considerations of planting density and spacing. Overcrowding could diminish the benefits of FR supplementation by increasing competition and reducing light penetration, particularly in dense planting arrangements (Jin *et al*., 2023). Therefore, optimizing light regimes must involve a holistic approach that considers not only the timing and intensity of FR exposure but also the spatial arrangement of plants to maximize overall system efficiency. The vertical space should also be considered since FR tends to elongate the lettuce and cause plants to reach closer to the lights. This suggests that vertical space should be considered when FR is supplemented or substituted in PFAL production. In small multilayer PFALs, the inclusion of FR may also alter air distribution by affecting plant architecture. This alteration in air distribution can influence canopy airflow and its effect was highlighted in computational fluid dynamics (CFD) simulations sensitive to crop drag coefficients (Kang *et al*., 2024).

It has also been demonstrated that as plant growth progresses light does not reach the lower part of the canopy, leading to senescence (Thomas and Stoddart, 1980; Boonman *et al*., 2006). Recent work showed that upward lighting from the base combined with top lighting to reach lower leaves could be applicable to retarding senescence of lower leaves as well as improvement of plant growth (Zhang *et al*., 2015; Joshi *et al*., 2017; Saengtharatip *et al*., 2021; Yamori *et al*., 2021). In high-density PFAL systems, where maximizing space is critical, the enhanced canopy expansion could lead to increased competition for light (Kozai, 2013; Miao *et al*., 2023), potentially necessitating adjustments in plant spacing to maintain high yield and quality. Thus, in plant factories, optimizing not only plant density but also the wavelength and direction of the irradiated light is recommended for successful cultivation.

### Conclusions and future research

Although leaves sometimes contain anthocyanins and other pigments, FR light is absorbed exclusively by chlorophyll. In terrestrial plants, this is chlorophyll *a*, and the absorption spectra of leaves and leaf canopies are well documented. Therefore, in studies analysing the effects of FR light it is crucial to consider both the emission spectra of the light source and the absorption spectra of leaves or leaf canopies, and to determine the experimental settings.

Future research may build on the findings of Zhen and Bugbee (2020) by considering a more targeted approach to the application of 700–750 nm FR light. Rather than substituting one FR photon for one PAR photon, it may be beneficial to tailor FR supplementation based on its relative absorptance and in consideration of species-specific responses and environmental conditions in PFAL systems. Given the lower absorptance of 700–715 nm and especially 715–750 nm FR light relative to PAR wavebands in our ‘Red Fire’ red leaf lettuce, thoughtful experimental design remains critical in photosynthesis studies. Moreover, more in-depth research into the mechanistic basis of FR is essential.

## Supplementary Material

mcag031_Supplementary_Data

## Data Availability

The data that support our paper can be requested by contacting the corresponding author.

## SUPPLEMENTARY DATA

Supplementary data are available at *Annals of Botany* online and consist of the following. Table S1: list of specific primer sets of the anthocyanin biosynthetic genes used for gene expression analysis. Figure S1: light distribution across the 1200 mm × 300 mm cultivation panel. Figure S2: tray spacing. Figure S3: leaf light absorptance of leaves. Figure S4: photosynthetic gas exchange measurements for 100 s under conditions without FR light, followed by a 100-s measurement with FR illumination.

## References

[mcag031-B1] Amelii A, Cutolo EA, Montepietra D, et al 2026. Photosynthesis under far-red light—evolutionary adaptations and bioengineering of light-harvesting complexes. FEBS Letters 600: 164–187. doi:10.1002/1873-3468.70191 41147650 PMC12834006

[mcag031-B2] ASTM Standard . 2020. ASTM G173-03: standard tables for reference solar spectral irradiances: direct normal and hemispherical on 37° tilted surface. West Conshohocken, PA, USA: ASTM International. https://www.nrel.gov/grid/solar-resource/spectra-am1.5.html, https://www.astm.org/g0173-03r20.html (November 2024, date last accessed).

[mcag031-B3] Barbieri F, Barbi S, Bertacchini A, Montorsi M. 2023. Combined effects of different LED light recipes and slow-release fertilizers on baby leaf lettuce growth for vertical farming: modeling through DoE. Applied Sciences 13: 8687. doi:10.3390/app13158687

[mcag031-B4] Boonman A, Anten NP, Dueck TA, et al 2006. Functional significance of shade-induced leaf senescence in dense canopies: an experimental test using transgenic tobacco. The American Naturalist 168: 597–607. doi:10.1086/508633 17080359

[mcag031-B5] Carpineti C, Meinen E, Van Ruijven J, et al 2024. Guidelines for cultivation in a vertical farm. Wageningen: Wageningen Plant Research. doi:10.18174/656035

[mcag031-B6] Chen Y, Li T, Yang Q, et al 2019. UVA radiation is beneficial for yield and quality of indoor cultivated lettuce. Frontiers in Plant Science 10: 1563. doi:10.3389/fpls.2019.01563 31867029 PMC6910135

[mcag031-B7] Chow WS, Melis A, Anderson J. 1990. Adjustments of photosystem stoichiometry in chloroplasts improve the quantum efficiency of photosynthesis. Proceedings of the National Academy of Sciences of the United States of America 87: 7502–7506. doi:10.1073/pnas.87.19.7502 11607105 PMC54775

[mcag031-B8] Evans JR . 1987. The relationship between electron transport components and photosynthetic capacity in pea leaves grown at different irradiances. Functional Plant Biology 14: 157–170. doi:10.1071/PP9870157

[mcag031-B9] Eylands N, Mattson N. 2023. Influence of far-red intensity during the seedling stage on biomass production and photomorphogenic characteristics in leafy greens under sole-source lighting. Horticulturae 9: 1100. doi:10.3390/horticulturae9101100

[mcag031-B10] Franklin KA . 2008. Shade avoidance. New Phytologist 179: 930–944. doi:10.1111/j.1469-8137.2008.02507.x 18537892

[mcag031-B11] Furuta H, Qu Y, Ishizuka D, Kawabata S, Sano T, Yamori W. 2025. A novel multilayer cultivation strategy improves light utilization and fruit quality in plant factories for tomato production. Front Hortic 4: 1633097. doi:10.3389/fhort.2025.1633097

[mcag031-B12] Gommers CMM, Visser EJW, Onge KRS, Voesenek LACJ, Pierik R. 2013. Shade tolerance: when growing tall is not an option. Trends in Plant Science 18: 65–71. doi:10.1016/j.tplants.2012.09.008 23084466

[mcag031-B13] Goto E, Hayashi K, Furuyama S, Hikosaka S, Ishigami Y. 2016. Effect of UV light on phytochemical accumulation and expression of anthocyanin biosynthesis genes in red leaf lettuce. Acta Horticulturae 1134: 179–186. doi:10.17660/ActaHortic.2016.1134.24

[mcag031-B14] Guo W, Zheng B, Duan T, Fukatsu T, Chapman S, Ninomiya S. 2017. EasyPCC: benchmark datasets and tools for high-throughput measurement of the plant canopy coverage ratio under field conditions. Sensors 17: 798. doi:10.3390/s17040798 28387746 PMC5422159

[mcag031-B15] Hayashi S, Levine CP, Yu W, et al 2024. Raising root zone temperature improves plant productivity and metabolites in hydroponic lettuce production. Frontiers in Plant Science 15: 1352331. doi:10.3389/fpls.2024.1352331 38689844 PMC11058216

[mcag031-B16] He R, Zhang Y, Song S, Su W, Hao Y, Liu H. 2021. UV-A and FR irradiation improves growth and nutritional properties of lettuce grown in an artificial light plant factory. Food Chemistry 345: 128727. doi:10.1016/j.foodchem.2020.128727 33307433

[mcag031-B17] Jeong SJ, Niu G, Zhen S. 2024. Far-red light and temperature interactively regulate plant growth and morphology of lettuce and basil. Environmental and Experimental Botany 218: 105589. doi:10.1016/j.envexpbot.2023.105589

[mcag031-B18] Jezek M, Allan AC, Jones JJ, Geilfus C-M. 2023. Why do plants blush when they are hungry? New Phytologist 239: 494–505. doi:10.1111/nph.18833 36810736

[mcag031-B19] Jin W, Ji Y, Larsen DH, Huang Y, Heuvelink E, Marcelis LFM. 2023. Gradually increasing light intensity during the growth period increases dry weight production compared to constant or gradually decreasing light intensity in lettuce. Scientia Horticulturae 311: 111807. doi:10.1016/j.scienta.2022.111807

[mcag031-B20] Joshi J, Zhang G, Shen S, Supaibulwatana K, Watanabe CK, Yamori W. 2017. A combination of downward lighting and supplemental upward lighting improves plant growth in a closed plant factory with artificial lighting. HortScience 52: 831–835. doi:10.21273/HORTSCI11822-17

[mcag031-B21] Kaiser E, Weerheim K, Schipper R, Dieleman JA. 2019. Partial replacement of red and blue by green light increases biomass and yield in tomato. Scientia Horticulturae 249: 271–279. doi:10.1016/j.scienta.2019.02.005

[mcag031-B22] Kaiser E, Kusuma P, Vialet-Chabrand S, et al 2024. Vertical farming goes dynamic: optimizing resource use efficiency, product quality, and energy costs. Frontiers in Science 2: 1411259. doi:10.3389/fsci.2024.1411259

[mcag031-B23] Kang L, Zhang Y, Kacira M, Van Hooff T. 2024. CFD simulation of air distributions in a small multi-layer vertical farm: impact of computational and physical parameters. Biosystems Engineering 243: 148–174. doi:10.1016/j.biosystemseng.2024.05.004

[mcag031-B24] Keller M, Arnink KJ, Hrazdina G. 1998. Interaction of nitrogen availability during bloom and light intensity during veraison. I. Effects on grapevine growth, fruit development, and ripening. American Journal of Enology and Viticulture 49: 333–340. doi:10.5344/ajev.1998.49.3.333

[mcag031-B25] Kelly N, Runkle ES. 2023. End-of-production ultraviolet A and blue light similarly increase lettuce coloration and phytochemical concentrations. HortScience 58: 525–531. doi:10.21273/HORTSCI17108-23

[mcag031-B26] Kelly N, Runkle ES. 2024. Dependence of far-red light on red and green light at increasing growth of lettuce. PLoS One 19: e0313084. doi:10.1371/journal.pone.0313084 39546482 PMC11567594

[mcag031-B27] Kinnunen P, Guillaume JHA, Taka M, et al 2020. Local food crop production can fulfil demand for less than one-third of the population. Nature Food 1: 229–237. doi:10.1038/s43016-020-0060-7

[mcag031-B28] Kono M, Terashima I. 2014. Long-term and short-term responses of the photosynthetic electron transport to fluctuating light. Journal of Photochemistry and Photobiology B: Biology 137: 89–99. doi:10.1016/j.jphotobiol.2014.02.016 24776379

[mcag031-B29] Kono M, Yamori W, Suzuki Y, Terashima I. 2017. Photoprotection of PSI by far-red light against the fluctuating light-induced photoinhibition in *Arabidopsis thaliana* and field-grown plants. Plant & Cell Physiology 58: 35–45. doi:10.1093/pcp/pcw215 28119424

[mcag031-B30] Kono M, Kawaguchi H, Mizusawa N, Yamori W, Suzuki Y, Terashima I. 2020. Far-red light accelerates photosynthesis in the low-light phases of fluctuating light. Plant & Cell Physiology 61: 192–202. doi:10.1093/pcp/pcz191 31617558

[mcag031-B31] Kopsell DA, Kopsell DE. 2008. Genetic and environmental factors affecting plant lutein/zeaxanthin. Agro Food Industry Hi-Tech 19: 44–46.

[mcag031-B32] Kozai T . 2013. Resource use efficiency of closed plant production system with artificial light: concept, estimation and application to plant factory. Proceedings of the Japan Academy, Series B 89: 447–461. doi:10.2183/pjab.89.447 PMC3881955 24334509

[mcag031-B33] Kramer DM, Avenson TJ, Edwards GE. 2004. Dynamic flexibility in the light reactions of photosynthesis governed by both electron and proton transfer reactions. Trends in Plant Science 9: 349–357. doi:10.1016/j.tplants.2004.05.001 15231280

[mcag031-B34] Kusuma P, Pattison PM, Bugbee B. 2020. From physics to fixtures to food: current and potential LED efficacy. Horticulture Research 7: 56. doi:10.1038/s41438-020-0283-7 32257242 PMC7105460

[mcag031-B35] Kusuma P, Bugbee B. 2023. On the contrasting morphological response to far-red at high and low photon fluxes. Frontiers in Plant Science 14: 1185622. doi:10.3389/fpls.2023.1185622 37332690 PMC10274578

[mcag031-B36] Laby RJ, Kincaid MS, Kim D, Gibson SI. 2000. The *Arabidopsis* sugar-insensitive mutants *sis4* and *sis5* are defective in abscisic acid synthesis and response. The Plant Journal 23: 587–596. doi:10.1046/j.1365-313x.2000.00833.x 10972885

[mcag031-B500] Lazzarin M, Dupont K, van Ieperen W, Marcelis LFM, Driever SM. 2025. Far-red light effects on plant photosynthesis: from short-term enhancements to long-term effects of artificial solar light. Annals of Botany 135:589–602. doi:10.1093/aob/mcae104 38946023 PMC11897601

[mcag031-B37] Levine C P, Hayashi S, Ohmori Y, et al 2023. Controlling root zone temperature improves plant growth and pigments in hydroponic lettuce. Annals of Botany 132: 455–470. doi:10.1093/aob/mcad127 37688538 PMC10667003

[mcag031-B38] Li Q, Kubota C. 2009. Effects of supplemental light quality on growth and phytochemicals of baby leaf lettuce. Environmental and Experimental Botany 67: 59–64. doi:10.1016/j.envexpbot.2009.06.011

[mcag031-B39] Li M, Jia N, Lenzen M, et al 2022. Global food-miles account for nearly 20 % of total food-systems emissions. Nature Food 3: 445–453. doi:10.1038/s43016-022-00531-w 37118044

[mcag031-B40] Li L, Sugita R, Yamaguchi K, Togawa H, Terashima I, Yamori W. 2025. High-precision lighting for plants: monochromatic red laser diodes outperform LEDs in photosynthesis and plant growth. Frontiers in Plant Science 16: 1589279. doi:10.3389/fpls.2025.1589279 40464012 PMC12129798

[mcag031-B41] Lichtenthaler HK . 1987. Chlorophylls and carotenoids: pigments of photosynthetic biomembranes. Methods in Enzymology 148: 350–382. doi:10.1016/0076-6879(87)48036-1

[mcag031-B42] Lim E, Kim J-O, Oh M-M. 2023. Optimizing the photon ratio of red, green, and blue LEDs for lettuce seedlings: a mixture design approach. Plant Methods 19: 121. doi:10.1186/s13007-023-01098-8 37926817 PMC10625695

[mcag031-B43] MathWorks . **2024.** *Regionprops function: measure properties of image regions*. https://www.mathworks.com/help/images/ref/regionprops.html (25 April 2025, date last accessed).

[mcag031-B44] Matsuura HN, De Costa F, Yendo ACA, Fett-Neto AG. 2013. Photoelicitation of bioactive secondary metabolites by ultraviolet radiation: mechanisms, strategies, and applications. In: Chandra S, Lata H, Varma A. eds. Biotechnology for medicinal plants. Berlin: Springer, 171–190. doi:10.1007/978-3-642-29974-2_7.

[mcag031-B45] Meng Q, Runkle ES. 2019. Far-red radiation interacts with relative and absolute blue and red photon flux densities to regulate growth, morphology, and pigmentation of lettuce and basil seedlings. Scientia Horticulturae 255: 269–280. doi:10.1016/j.scienta.2019.05.030

[mcag031-B46] Miao C, Yang S, Xu J, et al 2023. Effects of light intensity on growth and quality of lettuce and spinach cultivars in a plant factory. Plants 12: 3337. doi:10.3390/plants12183337 37765503 PMC10534974

[mcag031-B47] Ohtake N, Ishikura M, Suzuki H, Yamori W, Goto E. 2018. Continuous irradiation with alternating red and blue light enhances plant growth while keeping nutritional quality in lettuce. HortScience 53: 1804–1809. doi:10.21273/HORTSCI13469-18

[mcag031-B48] Ohtake N, Ju Y, Ishikura M, Suzuki H, Adachi S, Yamori W. 2021. Alternating red/blue light increases leaf thickness and mesophyll cell density in the early growth stage, improving photosynthesis and plant growth in lettuce. Environmental Control in Biology 59: 59–67. doi:10.2525/ecb.59.59

[mcag031-B49] Owen WG, Lopez RG. 2015. End-of-production supplemental lighting with red and blue light-emitting diodes (LEDs) influences red pigmentation of four lettuce varieties. HortScience 50: 676–684. doi:10.21273/HORTSCI.50.5.676

[mcag031-B50] Park Y, Runkle ES. 2017. Far-red radiation promotes growth of seedlings by increasing leaf expansion and whole-plant net assimilation. Environmental and Experimental Botany 136: 41–49. doi:10.1016/j.envexpbot.2016.12.013

[mcag031-B51] Pérez-Balibrea S, Moreno DA, García-Viguera C. 2008. Influence of light on health-promoting phytochemicals of broccoli sprouts. Journal of the Science of Food and Agriculture 88: 904–910. doi:10.1002/jsfa.3169

[mcag031-B52] Piovene C, Orsini F, Bosi S, et al 2015. Optimal red:blue ratio in led lighting for nutraceutical indoor horticulture. Scientia Horticulturae 193: 202–208. doi:10.1016/j.scienta.2015.07.015

[mcag031-B53] Qiu N, Shen H, Ishizuka D, et al 2025. Harnessing LED technology for consistent and nutritious production of large-fruited tomatoes. HortScience 60: 1851–1859. doi:10.21273/HORTSCI18868-25

[mcag031-B54] Qu Y, Sakoda K, Fukayama H, et al 2021. Overexpression of both Rubisco and Rubisco activase rescues rice photosynthesis and biomass under heat stress. Plant, Cell & Environment 44: 2308–2320. doi:10.1111/pce.14051 33745135

[mcag031-B55] Runkle E . **2022.** *Far-red light in greenhouse and indoor farming*. https://www.canr.msu.edu/floriculture/uploads/files/FR%20light.pdf (20 November 2024, date last accessed).

[mcag031-B56] Saengtharatip S, Joshi J, Zhang G, Takagaki M, Kozai T, Yamori W. 2021. Optimal light wavelength for a novel cultivation system with supplemental upward lighting in a plant factory with artificial lighting. Environment Control in Biology 59: 21–27. doi:10.2525/ecb.59.21

[mcag031-B57] Sager JC, Smith WO, Edwards JL, Cyr KL. 1988. Photosynthetic efficiency and phytochrome photoequilibria determination using spectral data. Transactions of the ASAE 31: 1882–1889. doi:10.13031/2013.30952

[mcag031-B58] Sasidharan R, Chinnappa CC, Voesenek LACJ, Pierik R. 2008. The regulation of cell wall extensibility during shade avoidance: a study using two contrasting ecotypes of *Stellaria longipes*. Plant Physiology 148: 1557–1569. doi:10.1104/pp.108.125518 18768908 PMC2577261

[mcag031-B59] Sharrock RA . 2008. The phytochrome red/far-red photoreceptor superfamily. Genome Biology 9: 230. doi:10.1186/gb-2008-9-8-230 18771590 PMC2575506

[mcag031-B60] Shikanai T . 2024. Molecular genetic dissection of the regulatory network of proton motive force in chloroplasts. Plant & Cell Physiology 65: 537–550. doi:10.1093/pcp/pcad157 38150384

[mcag031-B501] Shomali A, Kamrani YY, Zivcak M, Kovar M, Brestic M. 2025. Beyond Photosynthetic Active Radiation: The Role of Far-Red Energy and Signalling in the Improvement of Photosynthesis. Plant, Cell & Environment 48: 5009–5018. doi:10.1111/pce.15519 40148750

[mcag031-B502] Skabelund HA, Langenfeld NJ, Bugbee B. 2025. On the lack of morphological response to far-red at high and low photon flux in spinach. HortScience 60: 415–418. doi:10.21273/HORTSCI18410-24

[mcag031-B61] Stutte GW, Edney S, Skerritt T. 2009. Photoregulation of bioprotectant content of red leaf lettuce with light-emitting diodes. HortScience 44: 79–82. doi:10.21273/HORTSCI.44.1.79

[mcag031-B62] Suorsa M, Järvi S, Grieco M, et al 2012. PROTON GRADIENT REGULATION5 is essential for proper acclimation of *Arabidopsis* photosystem I to naturally and artificially fluctuating light conditions. The Plant Cell 24: 2934–2948. doi:10.1105/tpc.112.097162 22822205 PMC3426124

[mcag031-B63] Szymańska R, Ślesak I, Orzechowska A, Kruk J. 2017. Physiological and biochemical responses to high light and temperature stress in plants. Environmental and Experimental Botany 139: 165–177. doi:10.1016/j.envexpbot.2017.05.002

[mcag031-B64] Takano T, Wakabayashi Y, Wada S, Sano T, Kawabata S, Yamori W. 2025. Sustainable edamame production in an artificial light plant factory with improved yield and quality. Scientific Reports 15: 32083. doi:10.1038/s41598-025-17131-w 40940343 PMC12432140

[mcag031-B65] Taylor SH, Long SP. 2019. Phenotyping photosynthesis on the limit—a critical examination of RACiR. New Phytologist 221: 621–624. doi:10.1111/nph.15382 30198109

[mcag031-B66] Thilini Deepashika Perera WP, Navaratne S, Wickramasinghe I. 2022. Impact of spectral composition of light from light-emitting diodes (LEDs) on postharvest quality of vegetables: a review. Postharvest Biology and Technology 191: 111955. doi:10.1016/j.postharvbio.2022.111955

[mcag031-B67] Thomas H, Stoddart JL. 1980. Leaf senescence. Annual Review of Plant Physiology 31: 83–111. doi:10.1146/annurev.pp.31.060180.000503

[mcag031-B68] Tsormpatsidis E, Henbest RGC, Davis FJ, Battey NH, Hadley P, Wagstaffe A. 2008. UV irradiance as a major influence on growth, development and secondary products of commercial importance in Lollo Rosso lettuce Revolution’ grown under polyethylene films. Environmental and Experimental Botany 63: 232–239. doi:10.1016/j.envexpbot.2007.12.002

[mcag031-B69] Van Brenk JB, Courbier S, Kleijweg CL, Verdonk JC, Marcelis LFM. 2024. Paradise by the far-red light: far-red and red ratios independently affect yield, pigments, and carbohydrate production in lettuce (*Lactuca sativa*). Frontiers in Plant Science 15: 1383100. doi:10.3389/fpls.2024.1383100 38745919 PMC11091871

[mcag031-B70] Van De Velde E, Steppe K, Van Labeke M-C. 2023. Leaf morphology, optical characteristics and phytochemical traits of butterhead lettuce affected by increasing the far-red photon flux. Frontiers in Plant Science 14: 1129335. doi:10.3389/fpls.2023.1129335 37600174 PMC10433762

[mcag031-B71] Van Delden SH, SharathKumar M, Butturini M, et al 2021. Current status and future challenges in implementing and upscaling vertical farming systems. Nature Food 2: 944–956. doi:10.1038/s43016-021-00402-w 37118238

[mcag031-B72] Vergeer LHT, Aarts TL, De Groot JD. 1995. The ‘wasting disease’ and the effect of abiotic factors (light intensity, temperature, salinity) and infection with *Labyrinthula zosterae* on the phenolic content of *Zostera marina* shoots. Aquatic Botany 52: 35–44. doi:10.1016/0304-3770(95)00480-N

[mcag031-B73] Yamori W, Shikanai T. 2016. Physiological functions of cyclic electron transport around photosystem I in sustaining photosynthesis and plant growth. Annual Review of Plant Biology 67: 81–106. doi:10.1146/annurev-arplant-043015-112002 26927905

[mcag031-B74] Yamori W, Shikanai T, Makino A. 2015. Photosystem I cyclic electron flow via chloroplast NADH dehydrogenase-like complex performs a physiological role for photosynthesis at low light. Scientific Reports 5: 13908. doi:10.1038/srep13908 26358849 PMC4566099

[mcag031-B75] Yamori W, Makino A, Shikanai T. 2016a. A physiological role of cyclic electron transport around photosystem I in sustaining photosynthesis under fluctuating light in rice. Scientific Reports 6: 20147. doi:10.1038/srep20147 26832990 PMC4735858

[mcag031-B76] Yamori W, Kondo E, Sugiura D, Terashima I, Suzuki Y, Makino A. 2016b. Enhanced leaf photosynthesis as a target to increase grain yield: insights from transgenic rice lines with variable Rieske FeS protein content in the cytochrome b6/f complex. Plant, Cell & Environment 39: 80–87. doi:10.1111/pce.12594 26138548

[mcag031-B77] Yamori N, Matsushima Y, Yamori W. 2021. Upward LED lighting from the base suppresses senescence of lower leaves and promotes flowering in indoor rose management. HortScience 56: 716–721. doi:10.21273/HORTSCI15795-21

[mcag031-B503] Yoshiyama Y, Wakabayashi Y, Mercer KL, et al 2024. Natural genetic variation in dynamic photosynthesis is correlated with stomatal anatomical traits in diverse tomato species across geographical habitats. Journal of Experimental Botany 75: 6762–6777. doi:10.1093/jxb/erae082 38606772 PMC11639205

[mcag031-B78] Zhang G, Shen S, Takagaki M, Kozai T, Yamor W. 2015. Supplemental upward lighting from underneath to obtain higher marketable lettuce (*Lactuca sativa*) leaf fresh weight by retarding senescence of outer leaves. Frontiers in Plant Science 6: 1110. doi:10.3389/fpls.2015.01110 26697055 PMC4677251

[mcag031-B79] Zhen S, Bugbee B. 2020. Substituting far-red for traditionally defined photosynthetic photons results in equal canopy quantum yield for CO2 fixation and increased photon capture during long-term studies: implications for re-defining PAR. Frontiers in Plant Science 11: 581156. doi:10.3389/fpls.2020.581156 33014004 PMC7516038

[mcag031-B80] Zhu Y, Singh J, Patil BS, Zhen S. 2024a. End-of-production supplemental blue light intensity and duration co-regulate growth, anthocyanin, and ascorbic acid production in red leaf lettuce. Scientia Horticulturae 335: 113333. doi:10.1016/j.scienta.2024.113333

[mcag031-B81] Zhu Y, Wang H, Xiang X, et al 2024b. A dose-dependent effect of UV-328 on photosynthesis: exploring light harvesting and UV-B sensing mechanisms. Journal of Hazardous Materials 473: 134670. doi:10.1016/j.jhazmat.2024.134670 38781858

[mcag031-B82] Zou J, Zhang Y, Zhang Y, et al 2019. Morphological and physiological properties of indoor cultivated lettuce in response to additional far-red light. Scientia Horticulturae 257: 108725. doi:10.1016/j.scienta.2019.108725

[mcag031-B83] Zou J, Fanourakis D, Tsaniklidis G, Woltering EJ, Cheng R, Li T. 2023. Far-red radiation during indoor cultivation reduces lettuce nutraceutical quality and shortens the shelf-life when stored at supra optimal temperatures. Postharvest Biology and Technology 198: 112269. doi:10.1016/j.postharvbio.2023.112269

[mcag031-B84] Zou L, Huang JJ, Tan WK, et al 2024. Impact of different red/far-red light ratios on the morphological changes and nutritional profile of green leafy vegetable choy sum. ACS Food Science & Technology 4: 1052–1061. doi:10.1021/acsfoodscitech.3c00566

