# Supplementary material for: Far-red light in early growth stages boosts lettuce biomass and preserves anthocyanins
Source: Ann Bot. 2026 Mar 9;137(5):1215–32. doi: 10.1093/aob/mcag031 (PMC13197583; doi:10.1093/aob/mcag031)
Supplement: mcag031_Supplementary_Data [file mcag031_supplementary_data.zip › FigS1_V2_AOB-2025-483.pdf]

Figure S1.

PPFD (μmol m^{-2} s^{-1})

150 — 350

184	224	273	296	309	314	313	307	291	264	220	157
191	250	286	313	326	333	331	324	311	287	240	171
173	230	267	289	304	306	304	300	281	263	219	156

FR (μmol m^{-2} s^{-1})

45 — 100

64	76	89	94	98	99	96	96	89	78	70	47
59	73	82	88	91	93	92	91	89	82	68	49
59	76	84	91	97	98	98	97	90	87	74	54

3000 mm

1200 mm

Fig. S1 Light distribution across the 1200 mm x 300 mm cultivation panel, in of both PPFD and FR. Number and color indicated average photon density within 100 mm x 100 mm squares centered at corresponding locations on the panel. Plant materials were collected within area marked with dotted edges.
