# Supplementary material for: Far-red light in early growth stages boosts lettuce biomass and preserves anthocyanins
Source: Ann Bot. 2026 Mar 9;137(5):1215–32. doi: 10.1093/aob/mcag031 (PMC13197583; doi:10.1093/aob/mcag031)
Supplement: mcag031_Supplementary_Data [file mcag031_supplementary_data.zip › FigS3_V2_AOB_2025-483.pdf]

Figure S3.

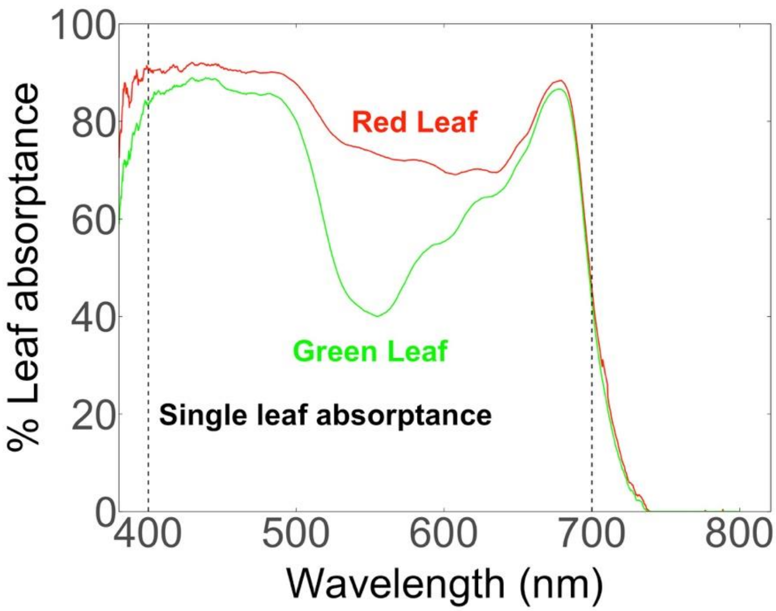

Fig. S3. Leaf light absorptance of two parts of leaves of red leaf lettuce. The space between the two dotted vertical lines indicates the PAR region (400-700 nm).
