# Supplementary material for: Far-red light in early growth stages boosts lettuce biomass and preserves anthocyanins
Source: Ann Bot. 2026 Mar 9;137(5):1215–32. doi: 10.1093/aob/mcag031 (PMC13197583; doi:10.1093/aob/mcag031)
Supplement: mcag031_Supplementary_Data [file mcag031_supplementary_data.zip › FigS4_V2_AOB-2025-483.pdf]

Figure S4.

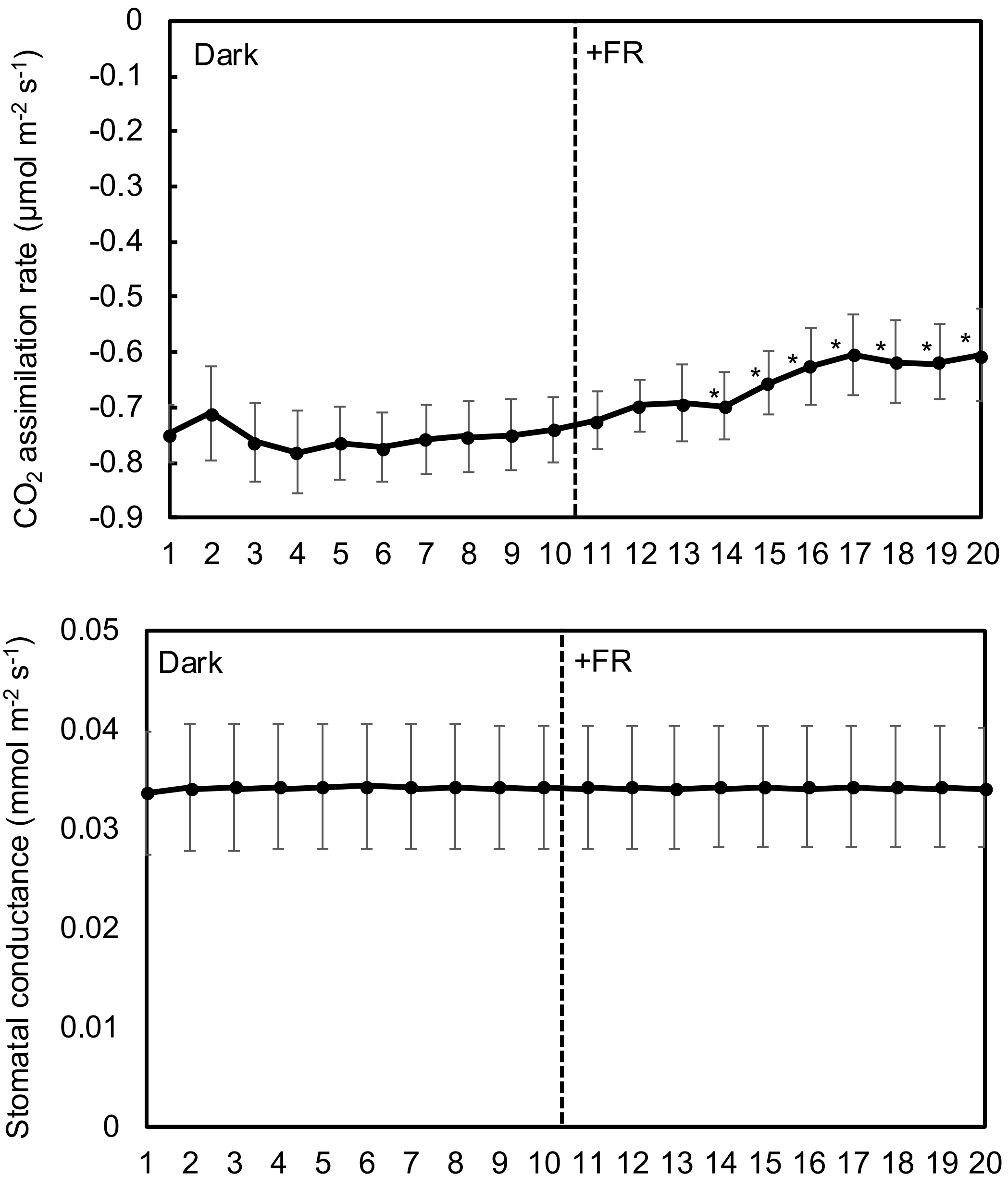

Fig. S4. Photosynthetic measurements were performed using fully expanded leaves of lettuce plants grown for one month. Gas exchange was first measured for 100 s under conditions without FR light, followed by a 100 s measurement with FR illumination. Because the FR light contained a small amount of PPFD, a slight increase in the CO_2 assimilation rate was observed, however, the effect was small. Therefore, under white-light conditions, the enhancement of CO_2 assimilation observed with FR illumination can be attributed mainly to the contribution of the FR component itself.
