# Supplementary material for: Far-red light in early growth stages boosts lettuce biomass and preserves anthocyanins
Source: Ann Bot. 2026 Mar 9;137(5):1215–32. doi: 10.1093/aob/mcag031 (PMC13197583; doi:10.1093/aob/mcag031)
Supplement: mcag031_Supplementary_Data [file mcag031_supplementary_data.zip › TableS1_AOB-2025-483.pdf]

Table S1. The specific primer sets of the anthocyanin biosynthetic genes used for the gene expression analysis.

Gene name	Accession number	Product length	Name	Primer sequence (5′−3′)
ACT	AB359898	113 bp	LsACT-F01	TGGTAGGTATGGGCCAGAAA
			LsACT-R01	GTCATCCCAGTTGCTCACAA
CHS	AB525909	169 bp	LsCHS-F02	GGAGGTGGGGCTAACTTTTC
			LsCHS-R02	GAGCTCCACCTGGTCCAATA
F3H	AB525910	210 bp	LsF3H-F02	CTACTCAAGGTGGCCCGATA
			LsF3H-R02	AATGTGAGATCGGGTTGAGG
DFR	CV700105	105 bp	LsDFR-F01	GGGAATGAGGGAGTGATGAA
			LsDFR-R01	ATTGGCAGAAAAAGCAFGCAT
ANS	AB525912	117 bp	LsANS-F01	CTCCCCACCATCGACTTAAA
			LsANS-R01	ATGGTTGACGAGATGCATGA
UFGT	AB525911	203 bp	LsUFGT-F02	AAGAGACCAGAACCCCGTTT
			LsUFGT-R02	AGCTCCAATGCTCTCCGATA
