# Supplementary figures and images for: Far-red light in early growth stages boosts lettuce biomass and preserves anthocyanins
Source: Ann Bot. 2026 Mar 9;137(5):1215–32. doi: 10.1093/aob/mcag031 (PMC13197583; doi:10.1093/aob/mcag031)

Figure S2.

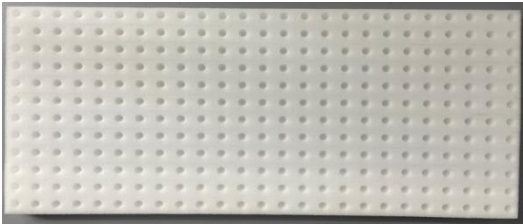

First 2 weeks

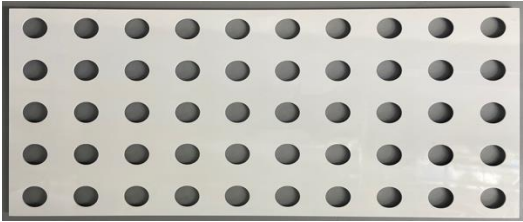

Next 2 weeks

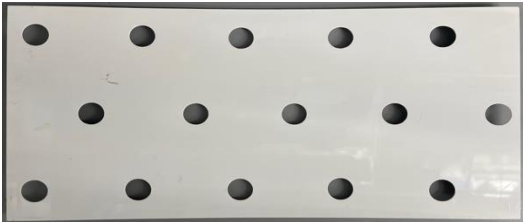

Last 2 weeks

Supplement: mcag031_Supplementary_Data [file mcag031_supplementary_data.zip › FigS2_V2_AOB-2025-483 .pdf]
